# Guidance for Canadian Breast Cancer Practice: National Consensus Recommendations for the Systemic Treatment of Patients with HR+/HER2− Early Breast Cancer 2025

**DOI:** 10.3390/curroncol33020112

**Published:** 2026-02-12

**Authors:** Sandeep Sehdev, Anil Abraham Joy, Jean-François Boileau, Nathaniel Bouganim, Christine Brezden-Masley, Jeffrey Q. Cao, David W. Cescon, Stephen Chia, Scott Edwards, Karen A. Gelmon, Katarzyna J. Jerzak, Aalok Kumar, Kara Laing, Nathalie LeVasseur, Christine Simmons, Marc Webster, Mita Manna, on behalf of Patient Advocacy, Breast Cancer Canada

**Affiliations:** 1 The Ottawa Hospital Cancer Centre, Ottawa, ON K1H 8L6, Canada; 2 Cross Cancer Institute, University of Alberta, Edmonton, AB T6G 1Z2, Canada; 3 Jewish General Hospital, McGill University, Montreal, QC H3T 1E2, Canada; jean-francois.boileau@mcgill.ca; 4 McGill University Health Centre, McGill University, Montreal, QC H4A 3J1, Canada; nathaniel.bouganim@mcgill.ca; 5 Marvelle Koffler Breast Centre, Mount Sinai Hospital, University of Toronto, Toronto, ON M5G 1X5, Canada; christine.brezden@sinaihealth.ca; 6 Arthur J.E. Child Comprehensive Cancer Centre, University of Calgary, Calgary, AB T2N 5G2, Canada; jeffrey.cao@albertahealthservices.ca (J.Q.C.); marc.webster@albertahealthservices.ca (M.W.); 7 Princess Margaret Cancer Centre, University of Toronto, Toronto, ON M5G 2M9, Canada; dave.cescon@uhn.ca; 8 BC Cancer—Vancouver, University of British Columbia, Vancouver, BC V5Z 4E6, Canada; schia@bccancer.bc.ca (S.C.); kgelmon@gmail.com (K.A.G.); nathalie.levasseur@bccancer.bc.ca (N.L.); christine.simmons@bccancer.bc.ca (C.S.); 9 Dr. H. Bliss Murphy Cancer Center, Memorial University of Newfoundland, St. John’s, NL A1B 3V6, Canada; scott.edwards@nlhealthservices.ca (S.E.); kara.laing@nlhealthservices.ca (K.L.); 10 Sunnybrook Odette Cancer Centre, University of Toronto, Toronto, ON M4N 3M5, Canada; katarzyna.jerzak@sunnybrook.ca; 11 BC Cancer—Surrey, University of British Columbia, Vancouver, BC V3V 1Z2, Canada; akumar7@bccancer.bc.ca; 12 Saskatoon Cancer Centre, University of Saskatchewan, Saskatoon, SK S7N 4H4, Canada

**Keywords:** hormone receptor-positive breast cancer, HER2-negative, early breast cancer, endocrine therapy, chemotherapy, CDK4/6 inhibitor, bisphosphonates, genomic testing, REAL Alliance, sentinel lymph node biopsy, axillary lymph node dissection

## Abstract

Hormone receptor-positive, human epidermal growth factor receptor 2-negative (HR+/HER2−) early breast cancer (EBC) is the most common breast cancer subtype and encompasses a biologically heterogeneous group of tumours. Endocrine therapy (ET) remains the cornerstone of treatment, but decisions regarding chemotherapy, cyclin-dependent kinase 4 and 6 (CDK4/6) inhibitors, and bone-modifying agents must be tailored to tumour biology, clinical stage, and menopausal status. REAL Canadian Breast Cancer Alliance (REAL Alliance), a pan-Canadian group of breast cancer specialists, convened to develop national clinical consensus recommendations for the systemic management of HR+/HER2− EBC. Using a structured consensus process, 28 recommendations were endorsed, spanning neoadjuvant and adjuvant systemic therapy, surgical considerations, and use of bisphosphonates. Key recommendations include the selective use of neoadjuvant chemotherapy for high-risk or locally advanced disease; genomic testing to guide chemotherapy decisions, particularly in postmenopausal patients; ET as the foundation of adjuvant therapy with intensification using CDK4/6 inhibitors in higher-risk patients; and adjuvant bisphosphonates in postmenopausal women to reduce recurrence and improve survival. These consensus recommendations provide practical, evidence-based guidance to support individualized, patient-centred management of HR+/HER2− EBC in the Canadian context.

## Simple Summary

Hormone receptor-positive, HER2-negative (HR+/HER2−) early breast cancer is the most common type of breast cancer and can vary widely in how it behaves and responds to treatment. While endocrine therapy is the foundation of care, additional treatments such as chemotherapy, targeted therapies, and bone-strengthening agents are beneficial for some patients but not others. To support consistent, evidence-informed decision-making across Canada, REAL Canadian Breast Cancer Alliance developed national consensus recommendations for the systemic treatment of HR+/HER2− early breast cancer. These recommendations address the use of treatments before and after surgery, incorporate tumour biology, stage, menopausal status, and patient preferences, and emphasize shared decision-making. The goal of this guidance is to help clinicians tailor treatment intensity to individual risk, avoid overtreatment where possible, and improve outcomes for patients across all provinces and territories.

## 1. Introduction

In countries with well-established breast cancer screening programmes, the majority of diagnoses occur at an early stage (stages I–III). Hormone receptor-positive, human epidermal growth factor receptor 2-negative (HR+/HER2−) disease accounts for approximately two-thirds of these cases, making it the most frequently encountered breast cancer subtype in routine practice [1,2,3,4]. In Canada alone, breast cancer remains a leading cause of cancer-related morbidity and mortality, with more than 30,000 new diagnoses and over 5000 deaths projected in 2024 [5], highlighting the ongoing need for optimized, evidence-based treatment strategies.

HR+/ HER2− early breast cancer (EBC) is biologically heterogeneous and treated with curative intent. Tumours range from luminal A to luminal B phenotypes, with less common basal or HER2-enriched subtypes identified only by gene expression profiling, which is not routinely used in clinical practice. Luminal A-like tumours, characterized by high estrogen receptor (ER) and/or progesterone receptor (PR) expression, low grade, and low proliferation, often achieve excellent outcomes on endocrine therapy (ET) alone. In contrast, luminal B-like tumours, defined genomically or by biological markers (e.g., high grade, elevated Ki-67, high genomic recurrence score, or low PR expression), carry higher recurrence risk and may benefit from chemotherapy or intensification of endocrine manipulation with therapies such as adjuvant cyclin-dependent kinase 4 and 6 inhibitors (CDK4/6is). Distinguishing patients who will derive meaningful benefit from treatment escalation from those who can safely avoid it remains a central clinical challenge, underscoring the need for practical, evidence-informed guidance.

Research Excellence, Active Leadership Canadian Breast Cancer Alliance (REAL Alliance) is a national, multidisciplinary collaboration of clinical–academic experts in breast cancer care, together with patient representation from Breast Cancer Canada. Established in December 2023, REAL Alliance was created to support the development of timely, evidence-based consensus recommendations aimed at promoting equitable access to high-quality breast cancer care across Canada. Through a series of disease-focused guidance documents, REAL Alliance seeks to inform clinical practice, policy development, and funding decisions using both robust clinical evidence and expert consensus.

Building on prior publications in this series, the present manuscript focuses on systemic therapy decision-making in HR+/HER2− EBC. The recommendations presented integrate tumour biology, clinical stage, menopausal status, and patient values to support shared decision-making and individualized treatment selection in contemporary practice.

## 2. Materials and Methods

### 2.1. Clinical Consensus Recommendation Process

The methodology used in this work was consistent with that of previous publications by REAL Alliance and is summarized here [6]. A targeted literature search was conducted to identify the evidence base relevant to the systemic treatment of HR+/HER2− EBC. Three members of REAL Alliance (S.S., A.A.J., J.-F.B.) acted as the sub-committee and identified the most relevant evidence. The sub-committee also developed the preliminary recommendations. The most relevant references were selected and linked to each recommendation. These recommendations were then subjected to a modified Delphi process in which an initial round of anonymous voting was carried out by an expert panel using an electronic platform. This panel consisted of 13 medical oncologists, 1 surgical oncologist, 1 radiation oncologist, 1 oncology pharmacist, and 1 patient advocacy representative, all with specialized expertise and extensive experience in managing breast cancer. During the voting process, panellists anonymously indicated their level of agreement with each statement, provided suggestions for revisions, and commented on specific references and background data.

The statements were then revised accordingly and discussed at a 2-day, in-person consensus meeting in Toronto, ON, Canada, in April 2025. During this meeting, the data were presented, and the wording of the recommendations was finalized. Following this meeting, a second round of anonymous electronic voting took place, where participants evaluated their agreement with each statement after reviewing a summary of the discussion and the corresponding references. A final round of edits was completed, and the third and final round of anonymous voting was conducted. A recommendation was considered accepted if more than 75% of the participants agreed with it after the predefined three rounds of voting. Consensus was reached on all 28 recommendations (Supplementary Materials—Voting Summary). The strength of recommendations was defined as follows: a strong recommendation is based on the highest quality of evidence; a moderate recommendation is supported by evidence that is less robust, indirect, or limited in scope; a weak recommendation is based on lower quality evidence; expert opinion reflects recommendations where there is endorsement by REAL Alliance, but for which there is limited evidence. As a final step, the sub-committee compared the current European Society for Medical Oncology (ESMO) and American Society of Clinical Oncology (ASCO) guidelines to REAL consensus recommendations.

### 2.2. Guiding Principles

At the outset of the development of these recommendations, REAL Alliance agreed that due to the biology of breast cancer, it is essential to use the most efficacious therapies for both early and advanced disease without consideration of access or reimbursement constraints, which change over time.

REAL Alliance recognizes the importance of health economic considerations, financial toxicity, and equitable access to care; however, the purpose of this guidance is to provide evidence-informed recommendations for best clinical practice, with the expectation that such consensus may help inform policy, funding, and implementation decisions across diverse Canadian healthcare settings.

Shared decision-making is implicit across all statements. While emphasized in specific recommendations where treatment choices are particularly nuanced, patient values and preferences should be incorporated into every stage of management.

Consideration of ovarian function suppression (OFS) is implied for all premenopausal women. For men with HR+ breast cancer, tamoxifen is the preferred ET, though an aromatase inhibitor (AI) + luteinizing hormone-releasing hormone (LHRH) agonist is an option. Men should otherwise be managed according to the same principles as pre/perimenopausal women, though caution is warranted when interpreting genomic assays such as Oncotype DX, which have not been extensively validated in male breast cancer.

ER-low tumours (1–10% ER expression) are biologically heterogeneous and may mirror triple-negative breast cancer (TNBC) with respect to clinical behaviour. Nonetheless, available data suggest that a subset of patients—particularly those with ER levels of 6–10%—may still derive benefit from ET. Until prospective evidence is available, ET should remain part of the discussion for these patients, with treatment decisions individualized according to tumour biology and patient preference.

The term “patient” is used throughout the consensus recommendations to refer to ciswomen and cismen with HR+/HER2− EBC. Recommendations for transgender patients must consider their exogenous endocrine maintenance therapy and ideally would be managed with multidisciplinary care, including an endocrinologist.

Though the statements are crafted to be as clear as possible, the reader is encouraged to read the accompanying narrative for the supporting evidence as well as any nuance associated with a given statement.

Finally, it is noteworthy that enrollment in clinical trials is always encouraged for patients who meet the clinical trial eligibility criteria.

## 3. Systemic Therapy in HR+/HER2− Early Breast Cancer

### 3.1. Neoadjuvant Treatment

Management of HR+/HER2− EBC requires a personalized approach that considers a patient’s clinical stage, tumour biology, and menopausal status. While ET forms the cornerstone of treatment, the role of chemotherapy, particularly in the neoadjuvant setting, varies significantly across patient subgroups. REAL Alliance recommendations, summarized in Table 1 and compared with ESMO and ASCO guidelines, describe the clinical scenarios in which neoadjuvant chemotherapy (NAC) or neoadjuvant ET (NET) may be appropriate, and when genomic testing may be used to guide these treatment decisions.

**Clinical Context**

In routine practice, especially for postmenopausal patients with operable disease, NAC plays a restricted role, as chemotherapy benefit is largely limited to those with high genomic risk or more advanced tumours. Nevertheless, NAC, and in select cases, NET, may have a strategic value where tumour or axillary downstaging could enable less extensive surgery. These decisions are often nuanced and frequently benefit from discussion at multidisciplinary tumour boards (MDT).

**Table 1:** Summary of REAL Alliance recommendations for the neoadjuvant treatment for HR+/HER2− EBC and comparison with those from ESMO and ASCO.

Recommendations for Neoadjuvant Treatment		REAL	ESMO	ASCO
**1**	**For patients with HR+/HER2− EBC where chemotherapy is clearly indicated and surgical information will not alter that decision,** NAC with shared decision-making should be considered.	Moderate recommendation ●●	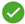	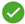
**2**	**For patients with HR+/HER2− EBC in whom the benefit of chemotherapy is uncertain, and where NAC is being considered through shared decision-making,** genomic recurrence risk testing on core biopsy should be considered to help guide treatment decisions.	Moderate recommendation ●●	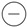 Genomic risk testing for the neoadjuvant setting is not covered	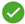
**3**	(a) **For premenopausal patients with T1–2, biopsy-proven N+, HR+/HER2− EBC,** NAC could be considered, especially in patients with high-risk features.	Weak recommendation ●	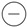 Decision based primarily on luminal A or B status	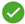
	(b) **For premenopausal patients with non-metastatic, inoperable T3–4 disease,** neoadjuvant systemic therapy is the standard of care and can potentially render patients with inoperable disease operable.	Strong recommendation ●●●		
**4**	(a) **For postmenopausal patients with T1–2, biopsy-proven N1, HR+/HER2− EBC,** upfront surgery is the standard of care.	Strong recommendation ●●●	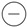 Decision based primarily on luminal A or B status	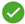
	**If neoadjuvant therapy is being considered,** genomic recurrence risk testing should be used to aid decision-making.	Moderate recommendation ●●		
	(b) **For postmenopausal patients with non-metastatic inoperable T3–4 disease,** neoadjuvant systemic therapy is the standard of care and can potentially render patients with inoperable disease operable.	Strong recommendation ●●●		
**5**	**For patients with cN2–3, HR+/HER2− EBC,** NAC is the standard of care, given its potential to downstage nodal disease and improve surgical options.	Strong recommendation ●●●	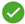	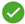
**6**	(a) **For patients with cN+, HR+/HER2− EBC where chemotherapy is indicated,** an anthracycline–taxane-based regimen is recommended in the neoadjuvant setting OR a taxane-based regimen +/− anthracycline is recommended in the adjuvant setting based on clinical risk, comorbidities, and shared decision-making.	Strong recommendation ●●●	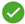	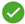
	(b) **For patients with cN+, HR+/HER2− EBC with cardiac or other contraindications to anthracyclines, concerns about long-term toxicity, and/or who decline anthracycline use after shared decision-making,** a non-anthracycline-based regimen (e.g., docetaxel + cyclophosphamide) is the standard of care.	Strong recommendation ●●●		
**7**	**For patients with inoperable, locally advanced, or inflammatory HR+/HER2− EBC who are appropriate candidates for chemotherapy,** NAC with an anthracycline–taxane-based regimen is the standard of care.	Strong recommendation ●●●	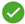	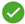
**8**	**For patients with inoperable breast cancer (potentially operable with downstaging) where chemotherapy is inappropriate or contraindicated**, NET is the standard of care with the goal of proceeding with surgery.	Strong recommendation ●●●	NC	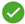
**9**	Routine use of neoadjuvant CDK4/6i + ET with curative intent is not recommended.	Strong recommendation ●●●	NC	NC
**10**	**For patients with HR+/HER2− EBC**, neoadjuvant immune checkpoint inhibition + chemotherapy is not currently recommended. This approach remains under active investigation for high-risk, high-grade disease.	Strong recommendation ●●●	NC	NC

●, Weak recommendation; ●●, Moderate recommendation; ●●●, Strong recommendation; 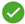, Alignment; 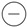, Some variation; NC, Not covered.

**Recommendation** **1:** *For patients with HR+/HER2− EBC where chemotherapy is clearly indicated and surgical information will not alter that decision, NAC with shared decision-making should be considered [Moderate recommendation].*

Patients with HR+/HER2− EBC for whom chemotherapy is clearly indicated are expected to derive a disease-free survival (DFS) and overall survival (OS) benefit [7,8,9,10,11]. This benefit is observed regardless of whether chemotherapy is administered in the neoadjuvant or adjuvant setting [12].

The rationale for offering chemotherapy in the neoadjuvant rather than adjuvant setting in HR+/HER2− disease is not to enhance long-term outcomes, but rather to achieve de-escalation of local therapies (i.e., surgery or radiotherapy). NAC can downstage tumours to increase the likelihood of breast-conserving surgery (BCS) and to enable sentinel lymph node biopsy (SLNB) or targeted axillary dissection (TAD) in place of axillary lymph node dissection (ALND) [13]. This approach is supported by landmark trials such as NSABP B-18 and B-27 [12,14,15], the European Cooperative Trial [16], and EORTC 10902 [17]. Across these studies, NAC consistently reduced tumour burden and improved rates of BCS without compromising survival. In these trials, up to 50% of patients achieved BCS after NAC.

Thus, unlike HER2-positive (HER2+) or triple-negative breast cancer (TNBC), where NAC is given to improve pathologic complete response (pCR) rates and improve prognosis, in HR+/HER2− EBC, the goal is to improve operability through axillary downstaging and possibly allow for less extensive surgical intervention [18].

As recommended by ASCO, ESMO, and the St. Gallen Consensus, NAC should be considered in selected patients for whom chemotherapy is clearly indicated based on clinical or biological risk [13,19,20]. In this context, clinical staging refers to assessment prior to definitive surgery based on physical examination, imaging, and core biopsy, and is distinct from pathologic staging determined after surgical resection, which informs postoperative management. These include patients with high-risk disease, such as those with cT3–T4 tumours (see Recommendations 3b and 4b), cN2–3 nodal involvement (see Recommendation 5), high-grade histology, or high genomic risk scores. REAL Alliance endorses these recommendations.

Importantly, while pathologic staging after surgery informs many aspects of adjuvant management (e.g., radiotherapy), nodal downstaging achieved with NAC should not be used to de-escalate eligibility for adjuvant systemic therapy escalation with CDK4/6is. Recommendations for adjuvant CDK4/6i use (see Recommendations 18, 20, and 21) should be based on the full extent of disease at presentation, including initial clinical stage and baseline tumour biology, rather than post-neoadjuvant pathologic findings.

As discussed in Recommendation 5, the likelihood of nodal and tumour response is higher in patients with luminal B-like tumours. Conversely, for patients with luminal A-like biology—characterized by high ER/PR expression, low grade, and low proliferation—NAC responsiveness is limited.

Finally, in patients for whom chemotherapy is clearly indicated, they may prefer chemotherapy in the neoadjuvant setting rather than the adjuvant setting to allow time for surgical decision-making, including consideration of BCS, mastectomy, and reconstruction options.

**Recommendation** **2:** *For patients with HR+/HER2− EBC in whom the benefit of chemotherapy is uncertain, and where NAC is being considered through shared decision-making, genomic recurrence risk testing on core biopsy should be considered to help guide treatment decisions [Moderate recommendation].*

Many patients with HR+/HER2− EBC fall into an intermediate-risk category—such as those with T1–2 tumours and cN1 disease—where the benefit of chemotherapy is uncertain. In these cases, gene expression assays such as Oncotype DX or MammaPrint may be considered to aid in treatment decision-making when NAC is being discussed.

These multigene assays were developed and validated using surgical specimens in the adjuvant setting to estimate recurrence risk and predict benefit from chemotherapy. Trials such as NSABP B-20 [21], TAILORx [22], RxPONDER [23], and MINDACT [24,25] established their prognostic and predictive utility in HR+/HER2− EBC (see Recommendation 4 for further discussion). However, their role in the neoadjuvant setting remains an area of evolving practice.

While prospective outcome data are limited, small studies and real-world evidence support the technical feasibility of performing genomic testing on core biopsy specimens, with high concordance between biopsy and surgical samples. A 2024 Canadian study demonstrated that Oncotype DX testing was technically feasible on core biopsy specimens in 95% of patients, with recurrence scores (RS) influencing treatment recommendations in nearly one-third of cases [26]. These findings are consistent with earlier studies reporting a high concordance for risk categorization using Oncotype DX on paired core and surgical specimens [27,28].

Nevertheless, it is important to note that these assays were not developed to guide treatment selection in the neoadjuvant setting. While high genomic risk may correlate with increased chemotherapy benefit in some patients, there is limited evidence supporting the use of these tools to predict pathologic response when treatment is given preoperatively. The ongoing NeoTAILOR trial (NCT05837455) is specifically evaluating whether Oncotype DX-guided chemotherapy in the neoadjuvant setting can impact residual disease and surgical outcomes in HR+/HER2− EBC [29].

The 2024 ESMO and 2022 ASCO guidelines acknowledge the use of gene expression profiling in patients with uncertain chemotherapy benefit, including in node-negative and select node-positive disease [19,30]. However, both guidelines stop short of endorsing testing on biopsy as a standard approach. The use of preoperative testing remains variable across institutions, and decisions should be individualized based on clinical risk, biopsy feasibility, and the potential for test results to alter management.

**Recommendation** **3:**

*(a)*  *For premenopausal patients with T1–2, biopsy-proven N+, HR+/HER2− EBC, NAC could be considered, especially in patients with high-risk features [Weak recommendation].*
*(b)*  *For premenopausal patients with non-metastatic, inoperable T3–T4 disease, neoadjuvant systemic therapy is the standard of care and can potentially render patients with inoperable disease operable [Strong recommendation].*

For premenopausal patients with T1–2, biopsy-confirmed N+, HR+/HER2− EBC, chemotherapy provides survival benefit regardless of timing, with long-term outcomes comparable between neoadjuvant and adjuvant administration. The advantage of neoadjuvant treatment is to facilitate surgical de-escalation, including potential downstaging of axillary disease [13]. However, that comes at the cost of potentially overtreating a patient with neoadjuvant anthracycline as compared to an adjuvant taxane-based regimen (see Recommendation 16). For those with larger tumours (T3–T4), NAC is the standard of care for the same reasons.

REAL Alliance considers NAC a reasonable strategy for patients with smaller tumours and low nodal burden but with high-risk features, such as luminal B-like tumours, due to their greater chemosensitivity. As noted in Recommendation 1, patients with clinical N+ disease may be eligible for SLNB or TAD instead of ALND if nodal response occurs with NAC.

In contrast, response rates to chemotherapy are generally low in luminal A-like tumours. Pathologic response is uncommon in these cases [18,31]. Residual cancer burden (RCB; score 0–3) can provide important prognostic information even in the absence of pCR. A large, international, pooled analysis of a cohort of 5161 patients with primary stage I–III breast cancer treated with NAC followed by surgery showed that higher RCB scores were significantly associated with worse event-free survival (EFS). However, the median follow-up of 56 months was relatively short, limiting confidence in longer-term results [32].

The role of genomic risk scores in guiding chemotherapy decisions for premenopausal women is limited. While these assays provide prognostic information on recurrence risk, they are less effective at predicting chemotherapy benefit, as demonstrated in the RxPONDER and MINDACT trials [23,24]. In both studies, younger women derived benefit from chemotherapy even when their genomic risk was low. For example, RxPONDER showed a 4.9% improvement in 5-year invasive DFS among premenopausal women with 1–3 positive nodes and RS ≤ 25 [23]. Similarly, MINDACT reported a 5.0% improvement in 8-year distant DFS for younger patients with high clinical but low genomic risk [25]. Overall, these findings indicate that treatment decisions should primarily focus on clinical and biological factors rather than genomic scores alone. However, most premenopausal patients in the RxPONDER study did not receive OFS, making it unclear if the benefit of chemotherapy was due to direct cytotoxic effects or treatment-induced menopause. The phase III OFSET (NRG-BR009) study evaluating OFS + ET compared to adjuvant chemotherapy + OFS + ET may help determine whether chemotherapy-induced amenorrhea or OFS affects recurrence risk in premenopausal patients with HR+, pN0–1 disease, and an Oncotype DX score ≤ 25 [33].

Growing evidence suggests that the observed benefit of chemotherapy in premenopausal women may be due to chemotherapy-induced amenorrhea. A 2022 meta-analysis concluded that amenorrhea after chemotherapy is associated with improved DFS and OS in premenopausal women, particularly those under 40 years of age [34]. This endocrine mechanism was not considered in the design of genomic trials, complicating the interpretation of chemotherapy benefits in younger women.

Given the importance of nodal status in guiding decisions, accurate nodal staging is critical. Suspicious nodes should undergo ultrasound-guided fine-needle aspiration, with clip placement in biopsy-confirmed nodes to enable TAD if nodal response is achieved.

**Recommendation** **4:**

*(a)*  *For postmenopausal patients with T1–2, biopsy-proven N1, HR+/HER2− EBC, upfront surgery is the standard of care [Strong recommendation]. If neoadjuvant therapy is being considered, genomic recurrence risk testing should be used to aid decision-making [Moderate recommendation].*
*(b)*  *For postmenopausal patients with non-metastatic, inoperable T3–4 disease, neoadjuvant systemic therapy is the standard of care and can potentially render patients with inoperable disease operable [Strong recommendation].*

For postmenopausal patients with T1–2, biopsy-confirmed N1, HR+/HER2− EBC, upfront surgery is the preferred approach. This reflects the modest responsiveness of HR+ tumours to chemotherapy and the lack of pCR as a validated surrogate for long-term outcomes in this subtype [13,18].

NAC may still be considered for patients with larger tumours (T3–4) or more aggressive biology (e.g., luminal B-like features), where surgical de-escalation may increase the likelihood of BCS and allow for SLNB or TAD in lieu of full ALND (see Recommendation 1).

In contrast to premenopausal patients, genomic risk scores play a more definitive role in guiding chemotherapy decisions in postmenopausal women. In RxPONDER and MINDACT, postmenopausal women with low to intermediate genomic risk (e.g., RS ≤ 25) derived no meaningful benefit from the addition of chemotherapy to ET [23,24]. In RxPONDER, postmenopausal women with 1–3 positive nodes and RS ≤ 25 experienced no improvement in invasive DFS with chemotherapy [23]. Similarly, the 8-year MINDACT analysis showed no distant DFS benefit with chemotherapy among women aged ≥ 50 years [25].

Conversely, chemotherapy may provide substantial benefit in postmenopausal women with high genomic risk. In a retrospective analysis of the NSABP B-20 trial, adding chemotherapy to tamoxifen in women with RS ≥ 25 improved 10-year distant DFS by 25.5% (88% vs. 62%; HR 0.27; 95% CI, 0.12–0.62), with a similar trend in women over age 50 (HR 0.44; 95% CI, 0.14–1.37) [35]. Together, these data reinforce the value of genomic profiling in guiding chemotherapy decision-making. The WSG ADAPT-HR+ study demonstrated that adaptive response decision-making using both dynamic biomarkers for early ET responsiveness (Ki-67 ≤ 10%) and static genomic profiling (RS) can inform therapeutic decisions to optimize therapy and help identify patients who can be safely treated by ET alone [36].

As discussed in Recommendation 2, genomic assays such as Oncotype DX were validated in the adjuvant setting, but studies have shown high concordance between core biopsies and resection specimens, supporting their use preoperatively when systemic treatment decisions are being made before surgery.

Thus, in postmenopausal women with luminal B-like tumours or ambiguous clinical risk, genomic testing on core biopsy can inform whether chemotherapy—and by extension, NAC—is warranted. However, in clinical practice, NAC is infrequently used in postmenopausal women with operable HR+/HER2− N1 disease, as chemotherapy benefit in this population is largely driven by high genomic risk. When NAC is considered in postmenopausal women, it is typically used for those with high genomic risk or those with locally advanced (T3–4) or borderline operable disease, or where NAC may allow for axillary surgical de-escalation. For patients in whom chemotherapy is less suitable, due to comorbidities, low-grade tumours, or classic lobular histology, NET may be considered (see Recommendations 10 and 11). Conversely, those with low genomic risk and limited nodal involvement may proceed directly to surgery.

Finally, ongoing studies such as Alliance A011202/MAC19 (NCT01901094) are exploring whether nodal surgery can be further de-escalated with NAC [37]. Until more definitive data are available, patients with residual nodal disease after NAC generally require ALND and/or regional nodal irradiation (RNI) [13].

**Recommendation** **5:** *For patients with cN2–3, HR+/HER2− EBC, NAC is the standard of care, given its potential to downstage nodal disease and improve surgical options [Strong recommendation].*

NAC is recommended for patients with HR+/HER2− EBC and clinically advanced nodal disease (cN2–3). This approach aligns with international guidelines that endorse NAC as the preferred strategy for this high-risk population, aiming to facilitate breast conservation and axillary downstaging (see Recommendation 1 for supporting evidence) [19,20]. As discussed in Recommendations 3 and 4, nodal response to NAC is more likely in tumours with luminal B-like features, while response is limited in luminal A-like or low–intermediate-grade lobular cancers.

Although pCR is uncommon in HR+/HER2− disease, evaluation of residual disease post NAC may inform prognosis and help identify patients who could benefit from additional adjuvant treatment.

Although NAC is standard in this setting, it may not be appropriate for all patients. In those with significant comorbidities, indolent tumour biology, or preferences against chemotherapy, endocrine-based approaches may be considered. These decisions require careful multidisciplinary input and shared decision-making.

**Recommendation** **6:**

*(a)*  *For patients with cN+, HR+/HER2− EBC, where chemotherapy is indicated, an anthracycline–taxane-based regimen is recommended in the neoadjuvant setting OR a taxane-based regimen +/− anthracycline is recommended in the adjuvant setting based on clinical risk, comorbidities, and shared decision-making [Strong recommendation].*
*(b)*  *For patients with cN+, HR+/HER2− EBC with cardiac or other contraindications to anthracyclines, concerns about long-term toxicity, and/or who decline anthracycline use after shared decision-making, a non-anthracycline-based regimen (e.g., docetaxel + cyclophosphamide) is the standard of care [Strong recommendation].*

As stated in Recommendation 1, when chemotherapy is clearly indicated in HR+/HER2− EBC, it may be delivered in either the neoadjuvant or adjuvant setting. Given the nuances in clinically node-positive disease, these decisions often benefit from MDT discussion. For patients at higher risk of recurrence—particularly those with node-positive disease—the preferred regimen in the neoadjuvant setting is an anthracycline–taxane-based combination (AC-T). The NSABP B-18 study demonstrated that neoadjuvant AC provides long-term survival outcomes equivalent to the same regimen given in the adjuvant setting [12,14]. NSABP B-27 further supported this neoadjuvant approach and showed that adding a taxane after AC significantly improved pCR rates and nodal downstaging compared to AC alone [15]. In the adjuvant setting, non-anthracycline regimens, such as TC × 4, are an acceptable alternative, particularly for patients with contraindications to anthracyclines. However, there is no robust evidence supporting the use of non-anthracycline regimens in the neoadjuvant setting. See Recommendation 16 for a full discussion of the evidence supporting anthracycline–taxane regimens and the role of TC in patients for whom anthracyclines are not an option.

**Recommendation** **7:** *For patients with inoperable, locally advanced, or inflammatory HR+/HER2− EBC who are appropriate candidates for chemotherapy, NAC with an anthracycline–taxane-based regimen is the standard of care [Strong recommendation].*

REAL Alliance endorses the ASCO guideline recommendation that NAC is the treatment of choice for patients with inoperable, locally advanced, or inflammatory breast cancer for whom a curative approach is appropriate [13]. In HR+/HER2− disease, where pCR is less common, the objective is to downstage the tumour, allowing for surgical resection with curative intent.

Although no modern randomized trials have focused exclusively on this subgroup, evidence from key studies such as NSABP B-18 and B-27 has demonstrated that NAC can convert inoperable disease to operable without compromising long-term outcomes [12,15]. Anthracycline–taxane-based regimens remain the preferred standard (see Recommendations 6 and 16).

**Recommendation** **8:** *For patients with inoperable breast cancer (potentially operable with downstaging) where chemotherapy is inappropriate or contraindicated, NET is a standard of care with the goal of proceeding with surgery [Strong recommendation].*

**Recommendation** **9:** *Routine use of neoadjuvant CDK4/6i + ET with curative intent is not recommended [Strong recommendation].*

NET is an established option in HR+/HER2− EBC when chemotherapy is unsuitable due to age, comorbidities, or patient preference. Its primary goal is tumour downstaging over 4–6 months to facilitate surgery. AIs are preferred, supported by trials such as P024, IMPACT, and ACOSOG Z1031, which demonstrated higher response and BCS rates compared with tamoxifen [38,39,40]. While NET achieves clinical responses comparable to chemotherapy, pCR rates remain low [41].

Adding a CDK4/6i to NET has improved biomarker endpoints in phase II studies (e.g., PALLET, NeoPalAna, neoMONARCH, FELINE, CORALLEEN), but no gains in radiologic, pathologic, or surgical outcomes have been shown [42,43,44,45,46]. A recent meta-analysis confirmed these findings [47]. Thus, CDK4/6i should not be used in the neoadjuvant curative setting outside clinical trials. In patients with inoperable disease who are not candidates for chemotherapy, ET with a CDK4/6i may be considered for non-curative intent disease control, drawing on evidence from the metastatic setting (e.g., MONALEESA-2, MONARCH-3) [48,49]. For additional guidance, readers are referred to the REAL Alliance recommendations for the treatment of metastatic breast cancer published in this issue.

In summary, NET is effective in carefully selected patients where chemotherapy is inappropriate, whereas neoadjuvant CDK4/6i should remain investigational or palliative.

**Recommendation** **10:** *For patients with HR+/HER2− EBC, neoadjuvant immune checkpoint inhibition + chemotherapy is not currently recommended. This approach remains under active investigation for high-risk, high-grade disease [Strong recommendation].*

The potential role of immune checkpoint inhibitors in HR+/HER2− EBC remains an area of ongoing investigation. Several randomized trials have demonstrated modest improvements in pCR with the addition of anti-programmed cell death protein 1 (PD-1) therapy to NAC, particularly in patients with high-risk disease. However, long-term outcome data are not yet mature, and pCR has not been validated as a surrogate for survival in this subtype.

The phase 2 I-SPY2 trial first established proof-of-concept for checkpoint inhibition in this setting [50]. In patients with high-risk HR+/HER2− tumours (as defined by MammaPrint), the addition of pembrolizumab to NAC more than doubled the estimated pCR rate compared to chemotherapy alone (30% vs. 13%). These findings provided the basis for subsequent phase 3 trials.

In the phase 3 KEYNOTE-756 trial, pembrolizumab plus NAC followed by adjuvant pembrolizumab and ET significantly improved pCR rates compared to placebo (24.3% vs. 15.6%) among patients with high-risk HR+/HER2− EBC [51]. pCR benefit was most pronounced in patients with higher programmed death-ligand 1 (PD-L1) expression (e.g., CPS ≥ 10 or ≥20) and those with ER positivity < 10%.

Similarly, the CheckMate 7FL trial (nivolumab plus NAC) met its primary endpoint [52]. A statistically significant increase in pCR was observed in the experimental arm (24.5% vs. 13.8%), with greater absolute benefit among patients with PD-L1-positive tumours (44.3% vs. 20.2%). Subgroup analyses also suggested enhanced benefit in patients with low ER and progesterone receptor expression and those with elevated stromal tumour-infiltrating lymphocytes (sTILs), though median follow-up is not yet sufficient for EFS outcomes.

While these trials establish pCR benefit in select biologically high-risk patients, the absolute improvements are modest. Use of neoadjuvant immune checkpoint inhibitors in HR+/HER2− EBC cannot be recommended until EFS outcomes are available.

**Figure 1:**
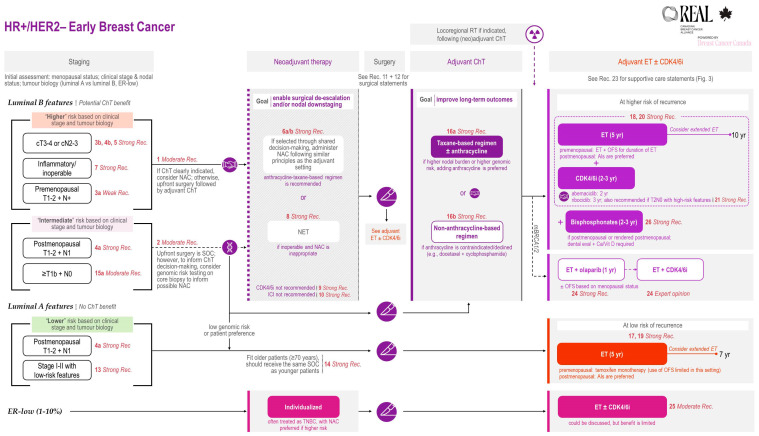
REAL Alliance recommendations for the management of HR+/HER2− EBC. AI = aromatase inhibitor; Ca = calcium; CDK4/6i = cyclin-dependent kinase 4/6 inhibitor; ChT = chemotherapy; cT = clinical tumour size; cN = clinical nodal status; ER = estrogen receptor; ET = endocrine therapy; HR+ = hormone receptor-positive; HER2− = human epidermal growth factor receptor 2-negative; NAC = neoadjuvant chemotherapy; NET = neoadjuvant endocrine therapy; OFS = ovarian function suppression; RT = radiotherapy; SOC, standard of care; TNBC = triple-negative breast cancer; Vit D = vitamin D; yr = year.

All REAL Alliance recommendations for NAC are outlined in an algorithm (see Figure 1).

### 3.2. Surgery

Surgical management of HR+/HER2− EBC has shifted toward less invasive approaches, guided by advances in systemic therapy and improved understanding of locoregional control. Decisions regarding the extent of axillary surgery, the use of clip placement, and the timing of definitive surgery must balance oncologic safety with long-term morbidity. The following REAL Alliance recommendations—summarized in Table 2 and compared with ESMO and ASCO guidelines—address contemporary approaches to axillary management, localization techniques, and surgical considerations in both younger and older patient populations.

**Recommendation** **11:**

*(a)*  *For patients with HR+/HER2− EBC and 1–2 positive sentinel nodes, performing further node dissection (e.g., ALND) to determine adjuvant systemic therapy is not recommended [Strong recommendation].*
*(b)*  *For patients with HR+/HER2− EBC and ≥3 positive sentinel nodes, MDT discussion (where available) regarding locoregional management is recommended [Strong recommendation].*

Axillary management has evolved substantially, with multiple trials showing that omission of ALND is safe in patients with limited sentinel node involvement. In ACOSOG Z0011, no survival or locoregional benefit was observed with completion ALND in patients undergoing BCS with 1–2 positive sentinel nodes [53]. The SENOMAC trial confirmed these findings in patients with sentinel node macrometastases [54], while AMAROS demonstrated equivalent control with axillary radiotherapy and lower morbidity compared with ALND [55].

While REAL Alliance does not recommend ALND in patients with 1–2 positive sentinel nodes, select cases may still warrant surgical evaluation, including those with breast cancer gene (*BRCA*) mutations eligible for adjuvant olaparib [56] or premenopausal women with extensive nodal disease, extranodal extension, or features qualifying them for adjuvant CDK4/6i [57]. Current approval criteria for abemaciclib include ≥4 positive nodes or 1–3 nodes with additional high-risk features [57]. Conversely, ALND would not be required for the use of adjuvant ribociclib for patients meeting NATALEE criteria [58]. Emerging approaches, such as TAD or those under investigation in the TAXIS trial, may further reduce morbidity while maintaining staging accuracy [59].

For patients with ≥3 positive sentinel nodes, the probability of additional nodal disease is high. In ACOSOG Z0011, 27% of patients with 1–2 positive sentinel nodes had additional non-sentinel involvement, suggesting even higher risk with ≥3 nodes [53]. The most recent ASCO Guidelines continue to recommend ALND for ≥3 nodes with the proviso that decision-making should be made on an individual case basis, incorporating a patient-centred approach respecting their values and preferences [60].

**Recommendation** **12:** *For patients with HR+/HER2− EBC where NAC is planned, placement of a clip to mark biopsied lesions in both breast and lymph nodes is the standard of care to aid surgical planning [Strong recommendation].*

As reported by REAL Alliance in 2024 in the *National Consensus Recommendations for Clinical Staging*, marking any biopsy-confirmed site of disease—whether in the breast or axilla—with a clip is recommended before starting neoadjuvant therapy [61]. This recommendation, along with other guidelines from Canadian experts [62], emphasizes the importance of clip placement not only for surgical localization and pathologic assessment but also for tailored approaches such as TAD and adjuvant radiotherapy decisions.

The response to NAC has direct implications for subsequent locoregional therapy. For patients with residual nodal disease, RNI or ALND may still be warranted, and accurate clip placement ensures treatment fields can be appropriately targeted. The A011202/MAC19 trial reported pathologic upstaging from N1 to N ≥ 2 in approximately 25% of patients [63]. In this setting, MDT discussion is essential to determine whether ALND or RNI is most appropriate, balancing oncologic benefit with long-term morbidity. The current standard of care generally favours ALND in this setting, though evolving practice patterns and individual patient characteristics may support alternative approaches. Shared decision-making and consideration of patient preferences are essential, given the changing landscape with additional radiologic and systemic adjuvant treatments available.

In contrast, for patients with biopsy-confirmed, node-positive disease who experience nodal pCR (ypN0) after NAC, the NRG-NSABP B-51/RTOG 1304 trial found no improvement in 5-year invasive breast cancer recurrence–free interval (IBCRFI) with the addition of RNI (92.7% vs. 91.8% without RNI; HR 0.88; 95% CI, 0.60–1.28) [64]. The pre-planned subgroup analysis according to stratification variables such as the type of surgery, hormone receptor or HER2 status, the presence or absence of a pCR in the breast, and adjuvant chemotherapy was consistent with the effect among the trial population overall. However, the most recent Postmastectomy Radiation Therapy: ASTRO-ASCO-SSO Clinical Practice Guideline notes conditionally that post-mastectomy radiation therapy may be omitted in the setting of pCR in both the breast and lymph nodes (ypT0N0) [65]. While most patients had HER2+ or TNBC, an exploratory analysis suggested a potential benefit of RNI in the HR+/HER2− subgroup (HR 0.41; 95% CI, 0.17–0.99), which requires confirmation with longer follow-up.

These principles align with earlier recommendations on the role of NAC in surgical downstaging (Recommendations 3 and 4) and axillary de-escalation (Recommendation 11).

**Recommendation** **13:** *For patients with clinical Stage I-II HR+/HER2− EBC with low-risk features, upfront surgery is the standard of care, with adjuvant systemic therapy guided by final pathology [Strong Recommendation].*

For patients with clinical Stage I–II HR+/HER2− EBC and low-risk features—such as node-negative status, low/intermediate grade, luminal A-like biology, and low genomic recurrence score—upfront surgery followed by adjuvant systemic therapy based on final pathology is the standard of care. NAC offers no survival advantage in this group and is not routinely indicated (see Recommendations 3, 4, and 15), unless required to facilitate breast conservation or downstage the axilla [13].

ET remains the primary adjuvant systemic treatment. Tamoxifen is preferred in premenopausal women, with consideration for OFS as the recurrence risk increases. In postmenopausal women, AIs—or sequential tamoxifen followed by AIs—are generally preferred over tamoxifen, though tamoxifen may be used if AIs are contraindicated or poorly tolerated (see Recommendations 17–20 for supporting evidence).

**Recommendation** **14:** *For patients aged ≥ 70 years with operable HR+/HER2− EBC who are fit for surgery, definitive surgery is the standard of care [Strong Recommendation].*

In fit women aged ≥ 70 with operable HR+/HER2− EBC, immediate surgery offers clear benefits over delaying or avoiding surgery and starting with ET. In a patient-level meta-analysis of three randomized trials (*n* = 1082), surgery plus tamoxifen compared with tamoxifen alone reduced 5-year locoregional recurrence from 45.8% to 12.1% (RR 0.24; 95% CI, 0.19–0.30) and was associated with lower risks of distant recurrence (RR 0.72; 95% CI, 0.57–0.90) and breast cancer mortality (RR 0.68; 95% CI, 0.54–0.86) in women aged ≥ 70 [66]. This study highlights the benefits of early surgical intervention, even in older women.

Surgery may be deferred in those with limited life expectancy, significant comorbidities, or high frailty scores, where primary ET is a reasonable option. The decision should be individualized through shared decision-making.

Guidance from Choosing Wisely Canada emphasizes that in older women with low-risk HR+/HER2− tumours, omission of SLNB and/or adjuvant radiotherapy may be appropriate after BCS, provided ET is administered [67]. ASCO’s 2025 update on SLNB use also supports this approach, stating that surgical axillary staging with SLNB is not recommended if the patient is clinically node-negative, without the explicit use of axillary ultrasound [60]. Education of both clinicians and patients remains key, as many older, fit individuals are still inappropriately deferred from surgical consultation.

**Figure 2:**
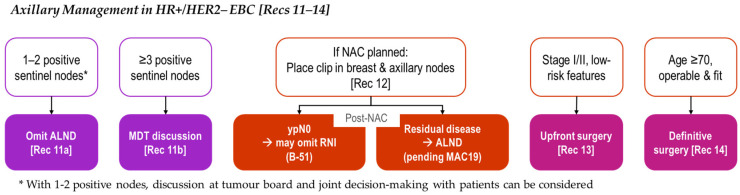
REAL Alliance recommendations for the axillary management of HR+/HER2− EBC. ALND = axillary lymph node dissection; MDT = multidisciplinary tumour board; NAC = neoadjuvant chemotherapy; RNI = regional nodal irradiation; ypN0 = pathologically node-negative after neoadjuvant therapy.

All REAL Alliance recommendations for the axillary management of HR+/HER2− EBC are outlined in Figure 2.

### 3.3. Adjuvant Chemotherapy

The role of adjuvant chemotherapy in HR+/HER2− EBC depends on tumour size, nodal status, genomic recurrence risk, and patient-specific factors. While ET remains the backbone of systemic treatment, chemotherapy provides incremental benefit in selected patients at higher risk of recurrence. The following REAL Alliance recommendations—summarized in Table 3 and compared with ESMO and ASCO guidelines—outline when genomic testing should guide adjuvant decision-making and highlight preferred chemotherapy regimens where treatment is indicated.

**Recommendation** **15:**

*(a)*  *For patients with ≥T1b, N0, HR+/HER2− EBC who are eligible for chemotherapy, and who have other intermediate- or high-risk features, genomic recurrence risk testing should be considered alongside shared decision-making, to determine the benefit of adjuvant chemotherapy [Moderate recommendation].*
*(b)*  *For patients with T1a, N0, HR+/HER2− EBC, there is insufficient data to support recurrence risk testing [Strong recommendation].*

REAL Alliance endorses ESMO, ASCO, and St. Gallen Consensus, all of which support the use of multigene expression assays to help guide adjuvant chemotherapy decisions in patients with HR+/HER2− EBC, particularly when clinical risk is intermediate or uncertain [19,20,30,68].

Genomic recurrence-risk testing is most appropriate for patients with HR+/HER2−, node-negative early breast cancers in whom the benefit of adjuvant chemotherapy is clinically uncertain based on standard pathologic features alone. Tumours that present with features suggesting a potentially higher baseline recurrence risk, yet not high enough to mandate chemotherapy outright, represent the group most likely to derive decision-making value from genomic testing. For patients with ≥T1b, node-negative HR+/HER2− disease, who have other intermediate- or high-risk features (e.g., Grade 2–3 histology, lymphovascular invasion [LVI], or elevated proliferative indices), genomic recurrence risk testing (e.g., Oncotype DX, MammaPrint) can provide prognostic and predictive information that complements traditional clinicopathologic features. Conversely, tumours that are very small (T1a) or have clearly low-risk biology (e.g., grade 1, low mitotic activity, or no LVI) generally have excellent outcomes with ET alone and are unlikely to benefit from genomic testing [22,24,35].

Emerging research suggests that further stratification may be possible. For instance, the MammaPrint ULTRA-LOW signature may identify a subset of patients with excellent long-term prognosis who could potentially reduce the duration of ET [69]. Additionally, ongoing studies are evaluating whether genomic assays can guide omission of adjuvant radiation in patients with very low recurrence scores and T1a tumours, although these strategies remain investigational.

Given the lack of definitive evidence in this group, recurrence risk testing should be undertaken only after shared decision-making, with a clear understanding of the limited expected benefit.

**Recommendation** **16:**

*(a)*  *For patients with cN+, HR+/HER2− EBC where chemotherapy is indicated and is being prescribed in the adjuvant setting, a taxane-based regimen +/− anthracycline is recommended based on clinical risk, comorbidities, and shared decision-making [Strong recommendation].*
*(b)*  *For patients with cN+, HR+/HER2− EBC with cardiac or other contraindications to anthracyclines, concerns about long-term toxicity, and/or who decline anthracycline use after shared decision-making, a non-anthracycline-based regimen (e.g., docetaxel + cyclophosphamide) is the standard of care [Strong recommendation].*

For patients with clinically node-positive HR+/HER2− EBC where chemotherapy is indicated (see Recommendation 1 for criteria), anthracycline–taxane-based regimens remain the standard of care. This approach is endorsed by both ASCO (2016, reaffirmed in subsequent updates) and ESMO (2024), which highlight their superior efficacy in reducing recurrence and breast cancer mortality, particularly in higher-risk patients [19,70,71,72]. Given the nuances of regimen selection in node-positive disease, these decisions often benefit from MDT discussion.

Evidence from randomized trials and meta-analyses supports this recommendation [7,8,9,10,11]. The 2023 Early Breast Cancer Trialists’ Collaborative Group (EBCTCG) patient-level meta-analysis, which included over 100,000 women across 86 trials, showed that adding an anthracycline to a taxane-based regimen reduced recurrence by 14% (RR 0.86; 95% CI, 0.79–0.93) and breast cancer mortality by 12% (RR 0.88; 95% CI, 0.78–0.99) over 10 years [11]. The most pronounced benefit was observed when anthracycline was added to docetaxel plus cyclophosphamide (TC): 10-year recurrence risk was reduced from 21.0% to 12.3% (absolute difference 8.7%; RR 0.58; 95% CI, 0.47–0.73), and mortality decreased by 4.2% (95% CI, 0.4–8.1).

In practice, several anthracycline-based regimens are used, including dose-dense AC (doxorubicin–cyclophosphamide) followed by paclitaxel (ddAC-P), AC–docetaxel (ddAC-D), and FEC (fluorouracil–epirubicin–cyclophosphamide)–docetaxel (FEC-D). Dose-dense strategies have shown improved DFS and OS. A 2019 EBCTCG analysis demonstrated that increasing dose intensity (e.g., dose-dense or sequential regimens) produced further reductions in recurrence (RR 0.86) and mortality (RR 0.87) [10].

Nevertheless, anthracycline-free regimens are appropriate alternatives in select patients. The PlanB and SUCCESS-C trials, along with earlier meta-analyses, showed comparable survival outcomes between TC × 6 and anthracycline–taxane regimens, especially in node-negative or lower-risk patients, with lower rates of certain toxicities, including mucositis, thrombocytopenia, and neuropathy [73,74,75]. The 2024 Canadian meta-analysis, which included 11,803 patients across seven randomized controlled trials (RCTs), confirmed little to no difference in DFS or OS between TC and anthracycline–taxane regimens [76]. However, subgroup analyses indicated that patients with ≥4 positive nodes derived a significant DFS benefit from anthracycline–taxane therapy over TC. Similarly, the pooled ABC trials found no OS difference but reported improved invasive DFS with anthracycline–taxane, particularly in node-positive disease [77]. Some real-world evidence suggests TC × 4 may offer similar efficacy to TC × 6, though prospective validation is limited [78]. In clinical practice, the number of TC cycles may vary by nodal burden and patient factors.

Genomic profiling may help identify patients most likely to benefit from anthracycline-containing regimens. A 2024 analysis of TAILORx (N0 patients) found that patients with RS > 31 and tumours ≥ 2 cm had better 5-year DFS with anthracycline–taxane chemotherapy versus TC (95.7% vs. 87.7%; HR 0.31) [79]. Likewise, in the FLEX registry, patients with Stage I–III MammaPrint High 2 Luminal B-like tumours had inferior 3-year outcomes when treated with TC compared to anthracycline-based regimens [80]. While these findings have not been prospectively evaluated, the data are noteworthy in providing caution against chemotherapy de-escalation approaches in this particularly high-risk group.

Together, these findings suggest that while TC is a reasonable and less toxic option for lower-risk or node-negative patients, anthracycline–taxane regimens remain preferred for those with higher nodal burden or higher genomic risk.

Anthracycline use must be weighed against the risk of serious toxicities, particularly cardiotoxicity. Cardiac dysfunction from anthracyclines is dose-dependent and can be irreversible, with the incidence of cardiac events estimated at ~5% at 400 mg/m^2^, rising sharply to 48% at 700 mg/m^2^ [81,82,83]. The risk increases with age and comorbidities, and is magnified by the use of AIs [84,85,86,87]. In a large, real-world cohort, anthracycline use was associated with an 82% increased risk of cardiomyopathy and/or heart failure compared to those who did not receive chemotherapy (HR 1.82; 95% CI, 1.47–2.25), while non-anthracycline chemotherapy was associated with a nonsignificant 18% increase (HR 1.18; 95% CI, 0.90–1.56) [88]. Importantly, asymptomatic cardiac dysfunction is likely more common and frequently underrecognized. Anthracycline cardiotoxicity is a progressive process, beginning with subclinical myocardial injury and evolving through stages of early left ventricular ejection fraction (LVEF) decline, increased ventricular wall thickness, and myofibrillar disarray [89]. Following anthracycline-based chemotherapy, long-term cardiac monitoring and proactive management of cardiovascular risk factors should be considered. In patients where the risk–benefit balance of anthracycline therapy is uncertain, referral to cardio-oncology can support individualized risk assessment and optimize management.

Furthermore, cardiovascular disease has surpassed breast cancer as the leading cause of death in survivors with pre-existing cardiac risk factors [90]. The CARDIAC-STAR study found that 61% of patients with newly diagnosed metastatic HR+/HER2− breast cancer had at least one cardiovascular comorbidity [91], suggesting that many early-stage patients may also warrant careful risk stratification.

The risk of therapy-related myelodysplastic syndrome (MDS) and acute myeloid leukemia (AML), though rare, adds to the cumulative risk profile and reinforces the need for long-term surveillance [9].

Ultimately, regimen selection should be guided by recurrence risk, comorbidities, and patient preferences and values. Anthracycline–taxane regimens remain the preferred standard for most patients with node-positive, HR+/HER2− EBC when chemotherapy is indicated. However, TC is an appropriate, evidence-based alternative in patients with contraindications or lower risk tolerance. Shared decision-making remains central to aligning treatment with patient-specific risks and goals.

### 3.4. Adjuvant Endocrine Therapy +/− CDK4/6 Inhibitor

ET is the cornerstone of adjuvant treatment for HR+/HER2− EBC, reducing recurrence and mortality across all stages. However, outcomes vary based on menopausal status, tumour biology, and genomic risk, and treatment intensification with CDK4/6is or bone-modifying agents may be warranted in higher-risk groups. The following REAL Alliance recommendations—summarized in Table 4 and compared with ESMO and ASCO guidelines—address patient selection, optimal ET backbones, integration of CDK4/6is, and the use of adjuvant bisphosphonates to further reduce recurrence risk.

**Recommendation** **17:** *For premenopausal women with N0, HR+/HER– EBC at low risk of recurrence, the standard of care treatment is ET [Strong recommendation].*

For premenopausal women with node-negative HR+/HER2− EBC who are at low risk of recurrence, five years of tamoxifen monotherapy remains the standard of care.

This recommendation is grounded in large randomized clinical trials and meta-analyses, including the 2011 EBCTCG meta-analysis, which demonstrated that 5 years of tamoxifen significantly reduced recurrences by ~50% during treatment and by ~30% over the first 10 years, and lowered breast cancer mortality by about one-third over 15 years in ER+ disease [92]. These benefits were consistent across subgroups, including by age, nodal status, tumour size, and grade—supporting the use of tamoxifen in premenopausal, low-risk patients.

Findings from the SOFT trial reinforce this approach. In the 12-year analysis, patients who did not receive chemotherapy—representing a clinically low-risk group—had excellent outcomes with tamoxifen alone [93]. This subgroup had favourable clinicopathologic features: 91% had node-negative disease, 86% had tumours ≤ 2 cm, and more than 90% had grade 1 or 2 histology. OS at 12 years exceeded 95%, with little difference observed between tamoxifen and more intensive ET arms.

While OFS may improve outcomes in higher-risk patients, its role in low-risk settings is limited. In patients with small, node-negative tumours—such as those not warranting chemotherapy—guidelines do not recommend the routine addition of OFS due to the modest benefit and greater risk of side effects, including vasomotor symptoms, sexual dysfunction, and bone loss [93].

The potential value of extended ET should also be considered. The ATLAS and aTTom trials demonstrated that continuing tamoxifen for 10 years further reduced recurrence and breast cancer mortality [94,95]. These trials included both pre- and postmenopausal women, and the relative benefit of extended therapy was consistent across subgroups. Based on these findings, and as summarized in ASCO’s guidelines, 7–8 years of total ET is generally appropriate for premenopausal patients with node-negative or limited nodal disease [96,97].

**Recommendation** **18:** For premenopausal women with HR+/HER2− EBC at high risk of recurrence *, the standard of care treatment is ET + CDK4/6i, as selected through shared decision-making [Strong recommendation].

(* as per the criteria of the monarchE and/or NATALEE trials)

This recommendation is consistent with international guideline endorsements, including those from ESMO and ASCO, for the use of adjuvant CDK4/6is in combination with ET in patients with HR+/HER2− EBC at high risk of recurrence [19,98]. CDK4/6i therapy represents a rational treatment intensification strategy in patients with high-risk clinicopathologic features, given the persistent risk of late recurrence in HR+ EBC and the limitations of ET [99].

“High-risk” disease is defined by the eligibility criteria used in the pivotal monarchE and NATALEE trials [58,100]. The monarchE trial enrolled patients with either ≥4 positive axillary lymph nodes or 1–3 positive nodes and one or more of the following features: tumour size ≥ 5 cm, histologic grade 3, or Ki-67 ≥ 20% [100]. The NATALEE trial included a broader patient population with stage II–III disease, including select patients with node-negative stage IIA disease and additional high-risk features (grade 3 histology, Ki-67 ≥ 20%, or high genomic risk) [58].

Both trials were designed and powered for invasive DFS as the primary endpoint, which is the most meaningful endpoint for patients in the adjuvant setting, according to Canadian patient survey data [101]. MonarchE was also powered for OS as a key secondary endpoint. NATALEE was not powered for OS, and thus, OS reports are exploratory.

Both monarchE and NATALEE demonstrated statistically significant and clinically meaningful improvements in invasive DFS. In monarchE, the 5-year interim analysis demonstrated that adding abemaciclib to ET improved invasive DFS by 7.6% (83.6% vs. 76.0%; HR 0.68; nominal *p* < 0.001), with sustained separation of the curves over time [100]. At the time of the interim analysis, 208 deaths (7.4%) had occurred in the abemaciclib + ET arm versus 234 deaths (8.3%) in the ET-alone arm (HR 0.903; 95% CI, 0.749–0.088; *p* = 0.284). In the updated 7-year analysis, invasive DFS improved by 6.5% (77.4% vs. 70.9%; HR 0.734; *p* < 0.0001), and OS improved by 1.8% (86.8% vs. 85.0%; HR 0.842; *p* = 0.0273) with abemaciclib + ET compared with ET alone [102]. In NATALEE, the protocol-specified final invasive DFS analysis (with median invasive DFS follow-up of 33.3 months) demonstrated a statistically significant improvement in invasive DFS with ribociclib added to a non-steroidal aromatase inhibitor (NSAI) compared to NSAI alone (HR 0.749; 95% CI, 0.628–0.892; two-sided *p* = 0.0012) [103]. In the 4-year landmark analysis, ribociclib + NSAI improved invasive DFS by 4.9% (88.5% vs. 83.6%; HR 0.715) [104] and improved 5-year invasive DFS by 4.5% (85.5% vs. 81.0%; HR 0.716; nominal *p* < 0.0001) compared to NSAI alone [105]. Though the OS data is still immature, there was a numerical trend in OS favouring ribociclib + NSAI (94.1% vs. 92.5%; HR 0.8; nominal *p* value 0.026). Of note, the high-risk, node-negative sub-group experienced a 5.7% absolute increase in 5-year invasive DFS with ribociclib versus NSAI alone (HR 0.606; 95% CI, 0.372–0.986). Clinical decision-making considers efficacy and safety data as well as patient preferences, comorbidities, and contraindications.

When choosing between abemaciclib and ribociclib for patients who meet eligibility for both trials, a few practical considerations may influence the decision [106,107]. These include treatment duration (2 years with abemaciclib vs. 3 years with ribociclib), maturity of follow-up data, and differences in toxicity profiles and monitoring requirements. Ribociclib is associated with a risk of QTc prolongation and requires baseline and early electrocardiogram (ECG) monitoring, as well as careful review of concomitant medications that may prolong QTc. In settings where ECG availability is limited, abemaciclib may be a more practical option. Conversely, abemaciclib is associated with higher rates of diarrhea and venous thromboembolism, particularly when combined with tamoxifen, and may be less suitable for patients with underlying gastrointestinal disorders or elevated thrombotic risk.

Endocrine partner selection may also influence CDK4/6i choice, as NATALEE permitted only NSAI, whereas monarchE allowed both AI and tamoxifen. Accordingly, the need for tamoxifen, such as intolerance to AI, may favour abemaciclib, while acknowledging the associated VTE risk. Additional factors, including patient comorbidities, dosing schedule (once daily for 3 weeks, followed by 1 week off vs. twice daily, continuously), and patient preferences, should be incorporated through shared decision-making. In the absence of head-to-head comparative data, CDK4/6i selection should be individualized based on clinical context, anticipated benefit, and treatment burden.

According to trial protocols, adjuvant abemaciclib should be initiated no later than 12 weeks after the initiation of ET and within 16 months of definitive surgery for early breast cancer [57]. For ribociclib, the protocol required that ribociclib treatment be started within 12 months of starting standard (neo)adjuvant ET and within 18 months of the initial diagnosis [58]. Both studies excluded the use of CDK4/6is during adjuvant radiotherapy to minimize toxicities, with initiation recommended at least two weeks following the completion of radiation.

For premenopausal women, ET should consist of an AI plus OFS, or tamoxifen plus OFS. The use of OFS in this population is supported by two large phase III trials—SOFT and TEXT—which demonstrated improved outcomes in premenopausal patients with HR+ EBC, particularly those at higher risk of recurrence [93]. In the SOFT trial, the addition of OFS to tamoxifen improved DFS compared with tamoxifen alone (76% vs. 72% at 12 years), with further improvement seen when exemestane was used instead of tamoxifen (DFS 79%; HR 0.69). The benefit of OFS was most pronounced in younger women and those who had received prior chemotherapy—features consistent with high-risk disease.

Further support for OFS comes from a 2023 patient-level EBCTCG meta-analysis [108]. Among premenopausal women, OFS significantly reduced the 15-year risk of recurrence by 12.1% (28.9% vs. 41.0%; RR 0.70), breast cancer mortality by 8.0% (20.9% vs. 28.9%; RR 0.69), and all-cause mortality by 7.2% (26.0% vs. 33.1%; RR 0.73), without increasing non-breast cancer mortality. The benefits were most pronounced in women under age 45.

Among premenopausal patients receiving OFS, an AI is generally preferred over tamoxifen in those with high-risk features. A joint analysis of SOFT and TEXT showed that exemestane plus OFS was associated with improved long-term outcomes compared to tamoxifen plus OFS, including a 3.3% absolute OS benefit at 12 years in patients who had received chemotherapy [109]. The benefit was more pronounced in high-risk subgroups (e.g., age < 35, grade 3 tumours, or tumour size > 2 cm). An EBCTCG meta-analysis of over 7000 women confirmed lower 10-year recurrence rates with AI versus tamoxifen (15% vs. 18%; RR 0.79), as well as improved distant recurrence rates (RR 0.83) [110].

Collectively, these data support the use of OFS plus an AI as the preferred endocrine backbone in premenopausal patients with high-risk HR+/HER2− EBC receiving adjuvant CDK4/6i therapy. The duration of OFS should mirror the full course of ET, which is typically at least five years. Based on trial designs, abemaciclib may be paired with either tamoxifen or an AI, although an AI is preferred given the higher risk of thromboembolism with tamoxifen, while ribociclib was paired with a NSAI to minimize QTc prolongation. In practice, REAL Alliance recommends a more flexible approach to AI selection, with the steroidal AI exemestane now permitted in trials such as Adjuvant WIDER (NCT05827081) [111].

**Recommendation** **19:** *For postmenopausal women with HR+/HER2− EBC at low risk of recurrence, the standard of care treatment is ET [Strong recommendation].*

ET is the mainstay of adjuvant treatment for postmenopausal women with HR+/HER2− EBC, even in those with low-risk clinical features. As outlined in Recommendation 20, tamoxifen has long been established as an effective therapy for reducing recurrence and mortality in HR+ disease. However, in postmenopausal women, AIs are preferred based on superior efficacy and long-term outcomes.

The 2015 EBCTCG meta-analysis, which included over 30,000 postmenopausal women, demonstrated that 5 years of an AI reduced the risk of breast cancer recurrence by 30% compared to tamoxifen (RR 0.70) and lowered 10-year breast cancer mortality by 15% (12.1% vs. 14.2%; RR 0.85) [112]. These benefits were consistent across subgroups regardless of nodal status, age, tumour grade, or HER2 status.

AIs may be given as monotherapy or as part of a sequential strategy, with 2–3 years of tamoxifen followed by an AI or vice versa to complete treatment. Sequential therapy is supported as a standard of care, both as an option for patients who cannot tolerate AIs and as a means to reduce cumulative AI-related toxicities [112]. This approach improves outcomes compared with 5 years of tamoxifen alone and yields outcomes comparable to AI monotherapy [112,113]. While tamoxifen alone remains acceptable for those who cannot tolerate AIs, the preferred standard is to incorporate an AI when feasible.

The duration of ET should be personalized. For most low-risk patients, 5 years is sufficient. However, tools such as Clinical Treatment Score post-5 years (CTS5), as well as clinical decision tools that incorporate genomic risk scores, such as the Breast Cancer Index (BCI) and RSClin Late, can estimate late recurrence risk and support decision-making about extending therapy beyond 5 years [114,115]. These tools help clinicians balance the potential benefits of extended therapy against toxicity and patient preferences.

In older or frail patients, shorter durations or even omission of ET may be appropriate based on shared decision-making and careful clinical judgement.

**Recommendation** **20:** For postmenopausal women with HR+/HER2− EBC at high risk of recurrence *, the standard of care treatment is adjuvant ET + CDK4/6i as selected through shared decision-making [Strong recommendation].

(* as per the criteria of the monarchE and/or NATALEE trials)

This recommendation aligns with international guidelines, including ASCO and ESMO, which support the use of adjuvant CDK4/6is for patients at high risk of recurrence [19,98]. As outlined in Recommendation 18, “high risk” includes patients who meet monarchE or NATALEE eligibility criteria [58,100]. In both trials, adding abemaciclib or ribociclib to ET significantly improved invasive DFS over ET alone.

For a review of the monarchE and NATALEE evidence supporting ET + CDK4/6i in this population, please see Recommendation 18.

ET in this population should consist of at least 5 years of an AI, either as monotherapy or following 2–3 years of tamoxifen [97]. As noted in Recommendation 20, the EBCTCG meta-analysis confirmed that AIs reduce recurrence and breast cancer mortality more effectively than tamoxifen [112].

Despite 5 years of ET, late recurrences are common in HR+ disease, especially in patients with greater initial tumour burden [116,117]. The EBCTCG Oxford overview showed that 20-year recurrence risk increases with stage, ranging from 10% to 57% depending on nodal status and tumour size [99]. More than half of recurrences occur after year 5.

For this reason, extended ET should be considered for high-risk patients. Randomized trials such as ATLAS [94], aTTom [95], and MA.17 [118] demonstrated that extending tamoxifen or introducing AI beyond 5 years reduces recurrences and breast cancer mortality. For patients treated initially with AI-based regimens, studies such as MA.17R [119], GIM-4 [120], ABCSG-16 [121], and an EBCTCG meta-analysis [122] suggest that the benefit of extended AI therapy is greatest in those with node-positive disease. In this group, a 5-year extension of AI therapy reduced the 15-year recurrence risk from 2.7% (node-negative to 3.8% node-positive) [122].

In clinical practice, 5 to 7 years of AI therapy is considered adequate for patients with low to intermediate risk (e.g., node-negative or 1–3 nodes), while up to 10 years may be warranted for patients with ≥4 positive nodes or other high-risk features [97,123]. As with initial ET selection, extended therapy decisions should consider patient age, bone health, side-effect burden, and adherence. Shared decision-making is essential to guide choices around therapy intensity, duration, and the use of CDK4/6is. Tools such as genomic assays and recurrence risk calculators may help individualize therapy and support informed patient choices.

**Recommendation** **21:** *For patients with T2N0 HR+/HER2− EBC and high-risk features (i.e., Ki-67 ≥ 20%, grade 3 histology, and/or high genomic risk score), the standard of care treatment is adjuvant AI + 3 years of ribociclib through shared decision-making [Strong recommendation].*

This recommendation addresses patients who are node-negative but have biologically aggressive tumours. These patients were included in the NATALEE trial, which enrolled individuals with T2N0 disease and at least one high-risk feature (Ki-67 ≥ 20%, grade 3 histology, or high genomic risk). In this subgroup (*n* = 285), adding ribociclib to a NSAI improved 4-year invasive DFS by 5.1% (92.1% vs. 87.0%; HR 0.666), suggesting consistent benefit across risk groups despite a lack of statistical power for subgroup significance [104]. In the updated analysis with 55.4 months of follow-up, adding ribociclib to NSAI improved 5-year invasive DFS by 5.7% (90.3% vs. 84.6%; HR 0.606) in the node-negative subgroup compared to NSAI alone [105].

While node-negative status often suggests a favourable prognosis, long-term data highlight persistent risk. In the Oxford overview, patients with T2N0 disease who received 5 years of ET had a 20-year recurrence risk of 29% [99]. A large real-world cohort similarly reported a 10-year recurrence risk of 37% in node-negative patients [124]. A real-world study estimated the risk of recurrence using the Flatiron database and determined that node-negative, high-risk patients had the same risk of recurrence as patients with N1 disease [125].

In this context, treatment escalation with ribociclib is recommended for selected high-risk T2N0 patients, such as those with Grade 3 disease, Ki-67 ≥ 20%, or high genomic risk. Shared decision-making is essential to weigh the benefits of therapy against potential side effects, cost, and patient preferences. The rationale for escalation should be clearly communicated, especially as patients may perceive node-negative disease as low risk.

In rare cases, NAC may be administered to patients initially staged as T2N0, most often due to high-risk biological or genomic features. In keeping with the NATALEE trial design and supported by REAL Alliance, post-neoadjuvant pathologic downstaging (e.g., ypT1cN0) should not be interpreted as indicating low baseline risk or used to de-escalate eligibility for adjuvant ribociclib. Decisions should instead be informed by the extent of disease and tumour biology at presentation, including pre-treatment grade, Ki-67, and genomic risk, and guided by shared decision-making and MDT discussion.

**Recommendation** **22:** *For patients with HR+/HER2− EBC who have been on ET + CDK4/6i for at least 6 months and are stable, clinical and laboratory monitoring can be done less frequently than monthly [Moderate recommendation].*

Most AEs with CDK4/6is occur early, typically within the first 2–3 months of therapy. According to product monographs, recommended monitoring includes every 2 weeks for the first 2 months, then monthly for 2–4 months (depending on the CDK4/6i), and then as clinically indicated. This schedule helps detect and manage AEs such as neutropenia, liver enzyme elevations, and diarrhea. Ribociclib requires ECG monitoring prior to initiation of therapy and then again on day 14 of cycle 1, and abemaciclib requires monitoring for and management of diarrhea [106,107].

For patients who tolerate the first 6 cycles well and are considered stable, clinicians may consider extended medication dispensing—one cycle plus refills for the next two cycles—accompanied by less frequent in-person visits. Importantly, even with a 3-month dispensing model, patients continue to have access to pharmacist or nursing consultation through alternative models of care, ensuring ongoing support, toxicity assessment, and adherence monitoring. (Please see recommendation 23) Such decisions should remain at the discretion of the treating physician, based on individual tolerance, adherence, and clinical judgement.

**Recommendation** **23:** *For patients with HR+/HER2− EBC receiving ET + CDK4/6i, management by an expert breast cancer healthcare professional practicing within an established monitoring pathway providing patient education, adherence support, drug/drug interaction assessment, side effect management, and blood work monitoring is the standard of care [Strong recommendation].*

Adjuvant ET plus CDK4/6is are most effective when taken consistently. However, treatment-related toxicities—particularly when patients cannot distinguish whether symptoms arise from ET or CDK4/6i—can lead to early discontinuation. In monarchE and NATALEE, ~19% of patients discontinued the CDK4/6i due to AEs.

Effective adherence support includes education about recurrence risk and treatment benefit, reassurance that side effects will be addressed promptly, symptom management strategies, and clear expectations about follow-up and dose adjustments [126].

Therefore, structured education and consistent follow-up are critical. The IMPACT trial demonstrated that a standardized patient coaching tool (MOATT) significantly improved 24-week persistence with abemaciclib plus ET, reducing permanent discontinuation by ~40% compared to usual care [127]. The TRADE study, also conducted with abemaciclib, used a dose-escalation strategy to improve tolerability [128]. All patients started abemaciclib at 50 mg twice daily for 2 weeks, escalated to 100 mg twice daily for 2 weeks, then escalated to the final dose of 150 mg twice daily onwards. Reduced incidence and severity of clinically important toxicity, such as diarrhea, were observed.

Multidisciplinary models—including medical oncologists, pharmacists, nurses, nurse practitioners, general practitioners in oncology (GPOs), and primary care providers—enhance adherence and patient satisfaction. These clinicians can contribute to medication education, adherence, lab monitoring, and toxicity management, especially important for older or more vulnerable patients [129].

Monitoring should follow product guidance: every 2 weeks for the first 2 months, then monthly until month 4–6, and as clinically indicated thereafter. Surveillance and patient support should be delivered within an established pathway led by clinicians experienced in breast cancer care.

**Recommendation** **24:** For patients with HR+/HER2− EBC and a germline BRCA1/2 pathogenic variant at high risk of recurrence *, the standard of care is olaparib for 1 year [Strong recommendation], followed by consideration for a CDK4/6i [Expert opinion].

(* as per the criteria of the OlympiA trial)

The OlympiA trial demonstrated that adjuvant olaparib significantly improved invasive DFS, distant DFS, and OS in patients with germline *BRCA1/2*-mutated, high-risk EBC, following standard local and systemic therapy. Patients met trial-defined risk criteria if they had either (1) residual disease after NAC with a clinical and pathological stage plus estrogen receptor and nuclear grade (CPS + EG) score ≥ 3 or (2) ≥4 pathologically confirmed positive lymph nodes after adjuvant chemotherapy. (The CPS+EG scoring system estimates relapse probability with scores ranging from 0 to 6, with higher scores indicating worse prognosis). One year of adjuvant olaparib improved invasive DFS (HR 0.63), distant DFS (HR 0.61), and OS (HR 0.68), regardless of HR status. At 4 years, the absolute benefits in invasive DFS, distant DFS, and OS were 7.3%, 7.4%, and 3.4%, respectively.

REAL Alliance recommends administering one year of olaparib in addition to standard ET for patients meeting criteria for the OlympiA trial.

Sequencing with CDK4/6is remains an open question. Current expert consensus, including the St. Gallen Consensus, supports sequential use—initiating olaparib first, followed by CDK4/6i if appropriate [20]. While prospective data are lacking, ongoing studies such as Adjuvant WIDER (NCT05827081) are exploring pragmatic sequencing strategies by allowing up to 36 months of ET prior to the initiation of ribociclib [111].

Thus, for patients with germline *BRCA1/2* mutations and high-risk HR+/HER2− disease, adjuvant olaparib represents the evidence-based standard. Sequential CDK4/6 inhibition in selected cases may be guided by multidisciplinary discussion and shared decision-making.

REAL Alliance supports the ASCO guidelines for germline testing in all newly diagnosed patients with breast cancer aged 65 years or younger [130]. For further guidance on BRCA testing, the reader is referred to Weber, E. et al. [131].

**Recommendation** **25:** *For patients with ER-low (1–10%) HR+/HER2− EBC, adjuvant ET +/− CDK4/6i could be discussed, though the absolute benefit is lower compared to more strongly ER+ tumours [Moderate recommendation].*

ER-low (1–10%) tumours are biologically heterogeneous and often behave more like triple-negative breast cancers (TNBC) than classic HR+ tumours. Current guidelines advise against managing ER-low tumours as traditional HR+ disease [19]. Indeed, they should be managed using TNBC algorithms.

Evidence supporting ET in this population is limited. Retrospective data suggest patients with ER 6–10% may derive modest benefit from ET, whereas ER 1–5% tumours show minimal response [132]. ET may still be offered, especially in the absence of competing treatment options, but expectations should be tempered.

In high-risk patients (e.g., grade 3 tumours, high Ki-67, node-positive), chemotherapy is generally recommended. Neoadjuvant therapy is preferred to allow response-guided adjuvant decisions. In stage II–III TNBC, pembrolizumab-based NAC (e.g., KEYNOTE-522 regimen) improved 3-year event-free survival (HR 0.63; 95% CI, 0.48–0.82) and remains the standard of care [133].

Notably, patients who receive pembrolizumab are not candidates for adjuvant CDK4/6is, but ET may still be discussed if ER ≥ 1%. For low-risk patients (e.g., pT1a/pN0), chemotherapy is generally not recommended, and ET may be offered selectively based on ER expression and patient values [19].

Shared decision-making is essential, as ER-low tumours occupy a grey zone between HR+ and TNBC biology. However, decisions should be driven by disease biology.

**Recommendation** **26:** *For postmenopausal women, or premenopausal women rendered postmenopausal, who are at higher risk of recurrence, bisphosphonates are the standard of care to reduce the risk of metastases [Strong recommendation].*

Adjuvant bisphosphonates reduce the risk of bone metastases and improve survival outcomes in postmenopausal women with HR+ EBC, particularly those with node-positive or otherwise high-risk disease. A 2015 EBCTCG meta-analysis showed that in postmenopausal women, bisphosphonates reduced bone metastases (HR 0.72), distant recurrence (HR 0.82), and breast cancer mortality (HR 0.82) [134]. This benefit was confirmed by individual trials such as AZURE (zoledronic acid), which reported improved invasive DFS among patients who were more than 5 years postmenopausal (HR 0.77). The class effect of bisphosphonates was also validated by SWOG S0307, showing no clear superiority of any specific agent [135].

Guidelines recommend initiating therapy within 2–3 months of surgery or chemotherapy [136,137]. Therapeutic options with the strongest supporting evidence include oral clodronate (1600 mg daily for 2–3 years) and intravenous zoledronic acid (either 4 mg every 6 months for 3 years or 4 mg every 3 months for 2 years, reflecting trial-based dosing regimens). Choice of bisphosphonate agent and regimen should consider patient preference, treatment accessibility, and jurisdictional availability.

The optimal duration of bisphosphonate therapy remains an area of clinical nuance. The SUCCESS-A trial suggested that treatment duration could potentially be shortened from the commonly recommended 3–5 years to 2 years [138].

Recent evidence suggests that the absolute benefit of adjuvant bisphosphonates may be smaller in patients receiving contemporary adjuvant systemic therapy (e.g., anthracyclines and taxanes), particularly among patients with higher-risk disease (e.g., multiple involved nodes, larger tumours, ER-negative status, or high-grade histology) [139]. Thus, careful discussion of risks, benefits, and individual preferences is warranted [140,141].

The adjuvant use of denosumab—an inhibitor of receptor activator of nuclear factor kappa-Β ligand (RANKL)—is currently uncertain. Although the ABCSG-18 trial showed a reduction in fracture risk (primary endpoint) and modest improvement in DFS with lower-dose denosumab [142], the D-CARE trial, using higher doses, did not show benefit in disease-related outcomes [143,144]. Consequently, denosumab is not routinely recommended for reducing recurrence risk at this time. If denosumab is being considered, the ABCSG-18 regimen should be favoured (i.e., postmenopausal HR+ women only, 60 mg every 6 months long term).

Patients should receive calcium and vitamin D, a dental assessment prior to starting therapy, and counselling on rare but serious risks such as osteonecrosis of the jaw [136,145]. Lifestyle counselling on smoking cessation, reduced alcohol intake, and exercise is also recommended [137].

**Figure 3:**
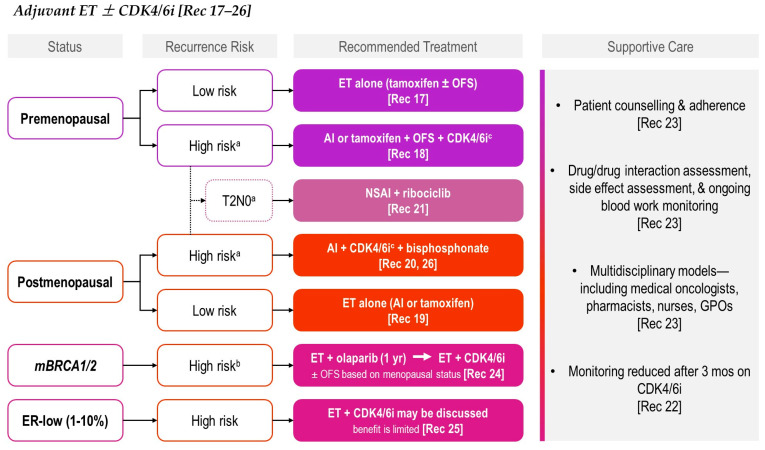
REAL Alliance recommendations for the adjuvant treatment of HR+/HER2− EBC. ^a^ High risk is defined according to trial criteria: for monarchE (abemaciclib), this included patients with either ≥4 positive axillary lymph nodes (ALNs) or 1–3 ALNs and ≥1 of the following features: tumour size ≥ 5 cm, grade 3 histology, or Ki-67 ≥ 20%; for NATALEE (ribociclib), this included all patients with Stage II–III, except for those with node-negative stage IIA disease who were required to have other high risk features (grade 3 histology, Ki-67 ≥ 20%, or high genomic risk). ^b^ Patients with HR+ disease were considered high-risk in OlympiA (olaparib) if they had (1) a clinical and pathological stage plus estrogen receptor and nuclear grade (CPS + EG) score of ≥3 after NAC, or (2) ≥4 positive nodes at initial surgery. ^c^ Abemaciclib may be combined with either tamoxifen or an AI; however, AI is generally preferred due to superior efficacy and the increased risk of thromboembolism associated with tamoxifen + abemaciclib. Ribociclib is paired with a NSAI to minimize QTc-prolongation risk. Abbreviations: AI = aromatase inhibitor; mBRCA1/2 = germline pathogenic breast cancer susceptibility gene 1/2 mutation; CDK4/6i = cyclin-dependent kinase 4/6 inhibitor; ET = endocrine therapy; ER = estrogen receptor; ER-low = estrogen receptor expression 1–10%; mos = months; NSAI = non-steroidal aromatase inhibitor; OFS = ovarian function suppression; T2N0 = clinical tumour size > 2 cm but ≤5 cm, node-negative; GPO = general practitioner in oncology.

All REAL Alliance recommendations for the management of EBC with adjuvant therapies are outlined in Figure 3.

### 3.5. Other Considerations

In addition to standard treatment decisions, REAL Alliance recognizes that certain patient groups require specific management considerations, including patients of childbearing potential and patients diagnosed during pregnancy (see Table 5).

**Recommendation** **27:** *For any patient of child-bearing potential with HR+/HER2− EBC, the standard of care is to discuss and provide information on family-planning and fertility-preservation options before treatment [Strong recommendation].*

REAL Alliance endorses the 2025 ASCO guideline update on fertility preservation, which recommends early discussions about infertility risk for all patients of childbearing potential prior to initiating treatment [146]. This includes not only chemotherapy, which poses the greatest risk, but also ET and OFS, which may affect fertility.

Fertility counselling should occur before treatment begins, with timely referral to a fertility specialist offered to those interested or uncertain. Pregnancy testing is also recommended prior to starting systemic therapy. These practices are supported by both ASCO and ESMO guidelines [19,146].

Notably, ESMO affirms that pregnancy after treatment is safe, reinforcing the value of proactive planning. Together, these guidelines establish a clear standard: fertility preservation should be offered early, before therapy begins, as part of comprehensive, patient-centred care.

**Recommendation** **28:** *For patients with HR+/HER2− EBC who are pregnant, consultation with relevant multidisciplinary specialists is the standard of care [Strong recommendation].*

The management of pregnancy-associated breast cancer is complex and requires careful coordination across disciplines to balance maternal cancer outcomes with fetal safety. A multidisciplinary approach involving oncology, maternal–fetal medicine, surgery, and neonatology is recommended [147,148,149].

Consistent with ASCO and ESMO guidance, chemotherapy is contraindicated during the first trimester due to risks of fetal malformations and miscarriage [147,148,150]. When clinically indicated, chemotherapy can be administered more safely during the second and third trimesters. Anthracyclines and taxanes have demonstrated relative safety in this setting, as supported by multiple studies [151,152,153,154,155,156,157]. However, dose-dense regimens are typically avoided because the required use of granulocyte-colony stimulating factor (G-CSF) lacks sufficient pregnancy safety data.

ETs are contraindicated throughout pregnancy due to teratogenic potential and limited safety data [158]. In a systematic review, major congenital malformations occurred in approximately 18% of live births following tamoxifen exposure during pregnancy [159]. However, evidence from the POSITIVE trial showed that temporary interruption of ET to attempt pregnancy did not increase breast cancer recurrence risk at 3 years (3-year incidence of 8.9% vs. 9.2% in matched controls from the SOFT/TEXT trial), suggesting that ET can be safely paused in selected patients wishing to conceive, provided that therapy is resumed postpartum [160]. The updated outcomes were presented at ESMO 2025 with a 71-month follow-up, confirming that temporary interruption of ET for pregnancy was not associated with worse breast cancer outcomes (5-year incidence of 12.3% vs. 13.2% in matched controls) [161].

Likewise, targeted agents and immunotherapies are not recommended in this context.

Supportive care should be carefully adapted. Methylprednisolone is preferred over dexamethasone to reduce fetal exposure, and ondansetron is considered a safe antiemetic. Histamine-2 receptor antagonists may be used for premedication with taxanes, while neurokinin-1 (NK1) receptor antagonists are generally avoided due to insufficient data [158].

If chemotherapy is administered, it is generally discontinued 2–3 weeks prior to the anticipated delivery date to reduce the risk of maternal or neonatal cytopenias [148].

Given the rarity and individualized nature of pregnancy-associated breast cancer, treatment decisions should be guided by gestational age, tumour biology, maternal preferences, and institutional expertise.

## 4. Conclusions

REAL Alliance consensus recommendations provide a practical framework for systemic treatment of HR+/HER2− EBC in Canada. ET remains the cornerstone of care, with chemotherapy, CDK4/6is, and bisphosphonates reserved for patients at higher risk. Treatment intensity should be tailored to clinical stage, tumour biology, and patient preference, while opportunities for surgical de-escalation are increasingly supported by systemic therapy advances.

Important gaps remain in areas such as ER-low tumours, the optimal duration of bisphosphonates, and the contribution of chemotherapy in premenopausal women beyond ovarian suppression effects. Ongoing trials and real-world evidence will help refine these questions.

This manuscript forms part of the continuing REAL Alliance series, which provides Canadian clinicians with timely, evidence-based guidance in areas of evolving practice.

## Figures and Tables

**Table 2:** Summary of REAL Alliance recommendations for surgery of HR+/HER2− EBC and comparison with those from ESMO and ASCO.

Recommendations for Surgery		REAL	ESMO	ASCO
**11**	(a) **For patients with HR+/HER2− EBC and 1–2 positive sentinel nodes,** performing further node dissection (e.g., ALND) to determine adjuvant systemic therapy is not recommended.	Strong recommendation ●●●	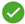	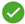
	(b) **For patients with HR+/HER2− EBC and ≥3 positive sentinel nodes,** MDT discussion (where available) regarding locoregional management is recommended.	Strong recommendation ●●●		
**12**	**For patients with HR+/HER2− EBC where NAC is planned,** placement of a clip to mark biopsied lesions in both breast and lymph nodes is the standard of care to aid surgical planning.	Strong recommendation ●●●	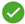	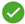
**13**	**For patients with clinical Stage I-II HR+/HER2− EBC with low-risk features,** upfront surgery is the standard of care, with adjuvant systemic therapy guided by final pathology.	Strong recommendation ●●●	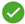	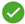
**14**	**For patients aged ≥ 70 years with operable HR+/HER2− EBC who are fit for surgery,** definitive surgery is the standard of care.	Strong recommendation ●●●	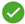	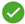

●●●, Strong recommendation; 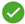, Alignment.

**Table 3:** Summary of REAL Alliance recommendations for adjuvant chemotherapy for HR+/HER2− EBC and comparison with those from ESMO and ASCO.

Recommendations for Adjuvant Chemotherapy		REAL	ESMO	ASCO
**15**	(a) **For patients with ≥T1bN0 HR+/HER2− EBC who are eligible for chemotherapy, and who have other intermediate- or high-risk features,** genomic recurrence risk testing should be considered alongside shared decision-making, to determine the benefit of adjuvant chemotherapy.	Moderate recommendation ●●	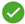	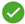
	(b) **For patients with T1a, N0, HR+/HER2− EBC**, recurrence risk testing should not be done.	Strong recommendation ●●●		
**16**	(a) **For patients with cN+, HR+/HER2− EBC where chemotherapy is indicated and is being prescribed in the adjuvant setting,** a taxane-based regimen +/− anthracycline is recommended based on clinical risk, comorbidities, and shared decision-making.	Strong recommendation ●●●	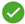	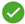
	(b) **For patients with cN+, HR+/HER2− EBC with cardiac or other contraindications to anthracyclines, concerns about long-term toxicity, and/or who decline anthracycline use after shared decision-making,** a non-anthracycline-based regimen (e.g., docetaxel + cyclophosphamide) is the standard of care.	Strong recommendation ●●●		

●●, Moderate recommendation; ●●●, Strong recommendation; 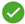, Alignment.

**Table 4:** Summary of REAL Alliance recommendations for adjuvant endocrine therapy +/− CDK4/6i for HR+/HER2− EBC and comparison with those from ESMO and ASCO.

Recommendations for Adjuvant ET +/− CDK4/6i		REAL	ESMO	ASCO
**17**	**For premenopausal women with N0, HR+/HER− EBC at low risk of recurrence,** the standard of care treatment is ET.	Strong recommendation ●●●	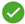	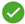
**18**	For premenopausal women with HR+/HER2− EBC at high risk of recurrence *, the standard of care treatment is adjuvant ET + CDK4/6i, as selected through shared decision-making. (* as per the criteria of the monarchE and/or NATALEE trials)	Strong recommendation ●●●	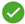	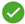
**19**	**For postmenopausal women with HR+/HER2− EBC at low risk of recurrence,** the standard of care treatment is ET.	Strong recommendation ●●●	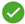	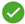
**20**	For postmenopausal women with HR+/HER2− EBC at high risk of recurrence *, the standard of care treatment is adjuvant ET + CDK4/6i as selected through shared decision-making. (* as per the criteria of the monarchE and/or NATALEE trials)	Strong recommendation ●●●	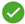	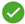
**21**	**For patients with T2N0, HR+/HER2− EBC and high-risk features (i.e., Ki-67 ≥ 20%, Grade 3 histology, and/or high genomic risk score),** the standard of care treatment is adjuvant AI + 3 years of ribociclib through shared decision-making.	Strong recommendation ●●●	NC	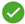
**22**	**For patients with HR+/HER2− EBC who have been on ET + CDK4/6i for at least 6 months and are stable,** clinical and laboratory monitoring can be done less frequently than monthly.	Moderate recommendation ●●	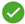	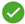
**23**	**For patients with HR+/HER2− EBC receiving ET + CDK4/6i,** management by an expert breast cancer healthcare professional practicing within an established monitoring pathway providing patient education, adherence support, drug/drug interaction assessment, side-effect management, and blood-work monitoring is the standard of care.	Strong recommendation ●●●	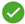	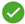
**24**	For patients with HR+/HER2− EBC and a germline *BRCA1/2* pathogenic variant at high risk of recurrence *, the standard of care treatment is olaparib for 1 year,	Strong recommendation ●●●	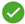	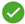
	followed by consideration for a CDK4/6i. (* as per the criteria of the OlympiA trial)	Expert opinion ○		
**25**	**For patients with ER-low (1–10%) HR+/HER2− EBC,** adjuvant ET +/− CDK4/6i * could be discussed, though the absolute benefit is lower compared to more strongly ER+ tumours. * *Use of CDK4/6i to be evaluated on case-by-case basis*	Moderate recommendation ●●	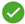	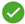 (Does not mention CDK4/6i)
**26**	**For postmenopausal women, or premenopausal women rendered postmenopausal, who are at higher risk of recurrence,** bisphosphonates are the standard of care to reduce the risk of metastases.	Strong recommendation ●●●	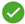	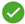

○, Expert opinion; ●●, Moderate recommendation; ●●●, Strong recommendation; 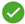, Alignment; NC, Not covered.

**Table 5:** Summary of REAL Alliance recommendations for fertility and pregnancy for HR+/HER2− EBC.

Recommendations for Fertility and Pregnancy		REAL	ESMO	ASCO
**27**	**For any patient of child-bearing potential with HR+/HER2− EBC**, the standard of care is to discuss and provide information on family-planning and fertility-preservation options before treatment.	Strong recommendation ●●●	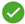	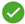
**28**	**For patients with HR+/HER2− EBC who are pregnant**, consultation with relevant multidisciplinary specialists is the standard of care.	Strong recommendation ●●●	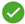	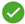

●●●, Strong recommendation; 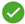, Alignment.

## Data Availability

No new data were generated.

## Supplementary Materials

The following supporting information can be downloaded at https://www.mdpi.com/article/10.3390/curroncol33020112/s1: Voting Summary.

## References

[1] Howlader N., Altekruse S.F., Li C.I., Chen V.W., Clarke C.A., Ries L.A.G., Cronin K.A. (2014). US Incidence of Breast Cancer Subtypes Defined by Joint Hormone Receptor and HER2 Status. J. Natl. Cancer Inst..

[2] Seung S.J., Traore A.N., Pourmirza B., Fathers K.E., Coombes M., Jerzak K.J. (2020). A Population-Based Analysis of Breast Cancer Incidence and Survival by Subtype in Ontario Women. Curr. Oncol..

[3] Cortet M., Bertaut A., Molinié F., Bara S., Beltjens F., Coutant C., Arveux P. (2018). Trends in Molecular Subtypes of Breast Cancer: Description of Incidence Rates between 2007 and 2012 from Three French Registries. BMC Cancer.

[4] Vaz-Gonçalves L., Marquart-Wilson L., Protani M.M., Stephensen M.T., Moore J., Morris M.F., Saunus J.M., Reeves M.M. (2025). Capturing Breast Cancer Subtypes in Cancer Registries: Insights into Real-World Incidence and Survival. J. Cancer Policy.

[5] Brenner D.R., Gillis J., Demers A.A., Ellison L.F., Billette J.-M., Zhang S.X., Liu J.L., Woods R.R., Finley C., Fitzgerald N. (2024). Projected Estimates of Cancer in Canada in 2024. CMAJ.

[6] Manna M., Gelmon K.A., Boileau J.-F., Brezden-Masley C., Cao J.Q., Jerzak K.J., Prakash I., Sehdev S., Simmons C., Bouganim N. (2024). Guidance for Canadian Breast Cancer Practice: National Consensus Recommendations for the Systemic Treatment of Patients with HER2+ Breast Cancer in Both the Early and Metastatic Setting. Curr. Oncol..

[7] Early Breast Cancer Trialists’ Collaborative Group (EBCTCG) (2005). Effects of Chemotherapy and Hormonal Therapy for Early Breast Cancer on Recurrence and 15-Year Survival: An Overview of the Randomised Trials. Lancet.

[8] Early Breast Cancer Trialists’ Collaborative Group (EBCTCG) (2008). Adjuvant Chemotherapy in Oestrogen-Receptor-Poor Breast Cancer: Patient-Level Meta-Analysis of Randomised Trials. Lancet.

[9] Early Breast Cancer Trialists’ Collaborative Group (Ebctcg) (2012). Comparisons between Different Polychemotherapy Regimens for Early Breast Cancer: Meta-Analyses of Long-Term Outcome among 100 000 Women in 123 Randomised Trials. Lancet.

[10] Gray R., Bradley R., Braybrooke J., Liu Z., Peto R., Davies L., Dodwell D., McGale P., Pan H., Taylor C. (2019). Increasing the Dose Intensity of Chemotherapy by More Frequent Administration or Sequential Scheduling: A Patient-Level Meta-Analysis of 37 298 Women with Early Breast Cancer in 26 Randomised Trials. Lancet.

[11] Braybrooke J., Bradley R., Gray R., Hills R.K., Pan H., Peto R., Dodwell D., McGale P., Taylor C., Aihara T. (2023). Anthracycline-Containing and Taxane-Containing Chemotherapy for Early-Stage Operable Breast Cancer: A Patient-Level Meta-Analysis of 100 000 Women from 86 Randomised Trials. Lancet.

[12] Fisher B., Brown A., Mamounas E., Wieand S., Robidoux A., Margolese R.G., Cruz A.B., Fisher E.R., Wickerham D.L., Wolmark N. (1997). Effect of Preoperative Chemotherapy on Local-Regional Disease in Women with Operable Breast Cancer: Findings from National Surgical Adjuvant Breast and Bowel Project B-18. J. Clin. Oncol..

[13] Korde L.A., Somer M.R., Hwang E.S., Khan S.A., Loibl S., Morris E.A., Perez A., Regan M.M., Spears P.A., Sudheendra P.K. (2021). Neoadjuvant Chemotherapy, Endocrine Therapy, and Targeted Therapy for Breast Cancer: ASCO Guideline. J. Clin. Oncol..

[14] Wolmark N., Wang J., Mamounas E., Bryant J., Fisher B. (2001). Preoperative Chemotherapy in Patients with Operable Breast Cancer: Nine-Year Results from National Surgical Adjuvant Breast and Bowel Project B-18. J. Natl. Cancer Inst. Monogr..

[15] Bear H.D., Anderson S., Brown A., Smith R., Mamounas E.P., Fisher B., Margolese R., Theoret H., Soran A., Wickerham D.L. (2003). The Effect on Tumor Response of Adding Sequential Preoperative Docetaxel to Preoperative Doxorubicin and Cyclophosphamide: Preliminary Results From National Surgical Adjuvant Breast and Bowel Project Protocol B-27. J. Clin. Oncol..

[16] Gianni L., Baselga J., Eiermann W., Guillem Porta V., Semiglazov V., Lluch A., Zambetti M., Sabadell D., Raab G., Llombart Cussac A. (2005). Feasibility and Tolerability of Sequential Doxorubicin/Paclitaxel Followed by Cyclophosphamide, Methotrexate, and Fluorouracil and Its Effects on Tumor Response as Preoperative Therapy. Clin. Cancer Res..

[17] van der Hage J.A., van de Velde C.J., Julien J.P., Tubiana-Hulin M., Vandervelden C., Duchateau L. (2001). Preoperative Chemotherapy in Primary Operable Breast Cancer: Results from the European Organization for Research and Treatment of Cancer Trial 10902. J. Clin. Oncol..

[18] Cortazar P., Zhang L., Untch M., Mehta K., Costantino J.P., Wolmark N., Bonnefoi H., Cameron D., Gianni L., Valagussa P. (2014). Pathological Complete Response and Long-Term Clinical Benefit in Breast Cancer: The CTNeoBC Pooled Analysis. Lancet.

[19] Loibl S., André F., Bachelot T., Barrios C.H., Bergh J., Burstein H.J., Cardoso M.J., Carey L.A., Dawood S., Del Mastro L. (2024). Early Breast Cancer: ESMO Clinical Practice Guideline for Diagnosis, Treatment and Follow-Up. Ann. Oncol..

[20] Curigliano G., Burstein H.J., Gnant M., Loibl S., Cameron D., Regan M.M., Denkert C., Poortmans P., Weber W.P., Thürlimann B. (2023). Understanding Breast Cancer Complexity to Improve Patient Outcomes: The St Gallen International Consensus Conference for the Primary Therapy of Individuals with Early Breast Cancer 2023. Ann. Oncol..

[21] Paik S., Tang G., Shak S., Kim C., Baker J., Kim W., Cronin M., Baehner F.L., Watson D., Bryant J. (2006). Gene Expression and Benefit of Chemotherapy in Women with Node-Negative, Estrogen Receptor-Positive Breast Cancer. J. Clin. Oncol..

[22] Sparano J.A., Gray R.J., Makower D.F., Pritchard K.I., Albain K.S., Hayes D.F., Geyer C.E., Dees E.C., Goetz M.P., Olson J.A. (2018). Adjuvant Chemotherapy Guided by a 21-Gene Expression Assay in Breast Cancer. N. Engl. J. Med..

[23] Kalinsky K., Barlow W.E., Gralow J.R., Meric-Bernstam F., Albain K.S., Hayes D.F., Lin N.U., Perez E.A., Goldstein L.J., Chia S.K.L. (2021). 21-Gene Assay to Inform Chemotherapy Benefit in Node-Positive Breast Cancer. N. Engl. J. Med..

[24] Cardoso F., van ’t Veer L.J., Bogaerts J., Slaets L., Viale G., Delaloge S., Pierga J.-Y., Brain E., Causeret S., DeLorenzi M. (2016). 70-Gene Signature as an Aid to Treatment Decisions in Early-Stage Breast Cancer. N. Engl. J. Med..

[25] Piccart M., van ’t Veer L.J., Poncet C., Lopes Cardozo J.M.N., Delaloge S., Pierga J.-Y., Vuylsteke P., Brain E., Vrijaldenhoven S., Neijenhuis P.A. (2021). 70-Gene Signature as an Aid for Treatment Decisions in Early Breast Cancer: Updated Results of the Phase 3 Randomised MINDACT Trial with an Exploratory Analysis by Age. Lancet Oncol..

[26] Luzhna L., Chia S., Pauls M., Levasseur N. (2024). Abstract PO3-15-04: Oncotype DX Recurrence Score as an Informative Tool to Optimize Neoadjuvant Therapy in HR-Positive, HER2-Negative Breast Cancers. Cancer Res..

[27] Orozco J.I.J., Chang S.-C., Matsuba C., Ensenyat-Mendez M., Grunkemeier G.L., Marzese D.M., Grumley J.G. (2021). Is the 21-Gene Recurrence Score on Core Needle Biopsy Equivalent to Surgical Specimen in Early-Stage Breast Cancer? A Comparison of Gene Expression Between Paired Core Needle Biopsy and Surgical Specimens. Ann. Surg. Oncol..

[28] Qi P., Yang Y., Bai Q., Xue T., Ren M., Yao Q., Yang W., Zhou X. (2021). Concordance of the 21-Gene Assay between Core Needle Biopsy and Resection Specimens in Early Breast Cancer Patients. Breast Cancer Res. Treat..

[29] Bagegni N., Summa T., Halim M., Grigsby I., Podany E., Luo J., Boulos F., Bennett D.L., Glover-Collins K., Olson J.A. (2025). Abstract P3-12-21: NeoTAILOR: A Phase II Biomarker-Directed Approach to Guide Neoadjuvant Therapy for Patients with Clinical Stage II/III ER+, HER2− Negative Breast Cancer. Clin. Cancer Res..

[30] Andre F., Ismaila N., Allison K.H., Barlow W.E., Collyar D.E., Damodaran S., Henry N.L., Jhaveri K., Kalinsky K., Kuderer N.M. (2022). Biomarkers for Adjuvant Endocrine and Chemotherapy in Early-Stage Breast Cancer: ASCO Guideline Update. J. Clin. Oncol..

[31] Guarneri V., Broglio K., Kau S.-W., Cristofanilli M., Buzdar A.U., Valero V., Buchholz T., Meric F., Middleton L., Hortobagyi G.N. (2006). Prognostic Value of Pathologic Complete Response after Primary Chemotherapy in Relation to Hormone Receptor Status and Other Factors. J. Clin. Oncol..

[32] Yau C., Osdoit M., Van Der Noordaa M., Shad S., Wei J., De Croze D., Hamy A.-S., Laé M., Reyal F., Sonke G.S. (2022). Residual Cancer Burden after Neoadjuvant Chemotherapy and Long-Term Survival Outcomes in Breast Cancer: A Multicentre Pooled Analysis of 5161 Patients. Lancet Oncol..

[33] Mamounas E.P., Tang G., Puhalla S.L., Swain S.M., Ganz P.A., Henry N.L., Cecchini R.S., Reid S.A., Rastogi P., Geyer C.E. (2025). A Phase III Trial Evaluating Addition of Adjuvant Chemotherapy to Ovarian Function Suppression + Endocrine Therapy in Premenopausal Women with pN0-1, HR+/HER2− Breast Cancer (BC) and Oncotype Recurrence Score (RS) ≤25 (OFSET): NRG-BR009. J. Clin. Oncol..

[34] Wang Y., Li Y., Liang J., Zhang N., Yang Q. (2022). Chemotherapy-Induced Amenorrhea and Its Prognostic Significance in Premenopausal Women With Breast Cancer: An Updated Meta-Analysis. Front. Oncol..

[35] Geyer C.E., Tang G., Mamounas E.P., Rastogi P., Paik S., Shak S., Baehner F.L., Crager M., Wickerham D.L., Costantino J.P. (2018). 21-Gene Assay as Predictor of Chemotherapy Benefit in HER2-Negative Breast Cancer. NPJ Breast Cancer.

[36] Nitz U.A., Gluz O., Kümmel S., Christgen M., Braun M., Aktas B., Lüdtke-Heckenkamp K., Forstbauer H., Grischke E.-M., Schumacher C. (2022). Endocrine Therapy Response and 21-Gene Expression Assay for Therapy Guidance in HR+/HER2– Early Breast Cancer. J. Clin. Oncol..

[37] (2025). A Randomized Phase III Trial Comparing Axillary Lymph Node Dissection to Axillary Radiation in Breast Cancer Patients (cT1-3 N1) Who Have Positive Sentinel Lymph Node Disease After Neoadjuvant Chemotherapy.

[38] Eiermann W., Paepke S., Appfelstaedt J., Llombart-Cussac A., Eremin J., Vinholes J., Mauriac L., Ellis M., Lassus M., Chaudri-Ross H.A. (2001). Preoperative Treatment of Postmenopausal Breast Cancer Patients with Letrozole: A Randomized Double-Blind Multicenter Study. Ann. Oncol..

[39] Smith I.E., Dowsett M., Ebbs S.R., Dixon J.M., Skene A., Blohmer J.-U., Ashley S.E., Francis S., Boeddinghaus I., Walsh G. (2005). Neoadjuvant Treatment of Postmenopausal Breast Cancer With Anastrozole, Tamoxifen, or Both in Combination: The Immediate Preoperative Anastrozole, Tamoxifen, or Combined With Tamoxifen (IMPACT) Multicenter Double-Blind Randomized Trial. J. Clin. Oncol..

[40] Ellis M.J., Suman V.J., Hoog J., Lin L., Snider J., Prat A., Parker J.S., Luo J., DeSchryver K., Allred D.C. (2011). Randomized Phase II Neoadjuvant Comparison Between Letrozole, Anastrozole, and Exemestane for Postmenopausal Women with Estrogen Receptor–Rich Stage 2 to 3 Breast Cancer: Clinical and Biomarker Outcomes and Predictive Value of the Baseline PAM50-Based Intrinsic Subtype—ACOSOG Z1031. J. Clin. Oncol..

[41] Sella T., Weiss A., Mittendorf E.A., King T.A., Pilewskie M., Giuliano A.E., Metzger-Filho O. (2021). Neoadjuvant Endocrine Therapy in Clinical Practice: A Review. JAMA Oncol..

[42] Johnston S., Puhalla S., Wheatley D., Ring A., Barry P., Holcombe C., Boileau J.F., Provencher L., Robidoux A., Rimawi M. (2019). Randomized Phase II Study Evaluating Palbociclib in Addition to Letrozole as Neoadjuvant Therapy in Estrogen Receptor–Positive Early Breast Cancer: PALLET Trial. J. Clin. Oncol..

[43] Ma C.X., Gao F., Luo J., Northfelt D.W., Goetz M., Forero A., Hoog J., Naughton M., Ademuyiwa F., Suresh R. (2017). NeoPalAna: Neoadjuvant Palbociclib, a Cyclin-Dependent Kinase 4/6 Inhibitor, and Anastrozole for Clinical Stage 2 or 3 Estrogen Receptor–Positive Breast Cancer. Clin. Cancer Res..

[44] Hurvitz S.A., Martin M., Press M.F., Chan D., Fernandez-Abad M., Petru E., Rostorfer R., Guarneri V., Huang C.-S., Barriga S. (2020). Potent Cell-Cycle Inhibition and Upregulation of Immune Response with Abemaciclib and Anastrozole in neoMONARCH, Phase II Neoadjuvant Study in HR+/HER2− Breast Cancer. Clin. Cancer Res..

[45] Khan Q.J., O’Dea A., Bardia A., Kalinsky K., Wisinski K.B., O’Regan R., Yuan Y., Ma C.X., Jahanzeb M., Trivedi M.S. (2020). Letrozole + Ribociclib versus Letrozole + Placebo as Neoadjuvant Therapy for ER+ Breast Cancer (FELINE Trial). J. Clin. Oncol..

[46] Prat A., Saura C., Pascual T., Hernando C., Muñoz M., Paré L., Farré B.G., Fernández P.L., Galván P., Chic N. (2020). Ribociclib plus Letrozole versus Chemotherapy for Postmenopausal Women with Hormone Receptor-Positive, HER2-Negative, Luminal B Breast Cancer (CORALLEEN): An Open-Label, Multicentre, Randomised, Phase 2 Trial. Lancet Oncol..

[47] Zhang Z., Zhao X., Chen J. (2024). Adjuvant and Neoadjuvant Therapy with or without CDK4/6 Inhibitors in HR+/HER2− Early Breast Cancer: A Systematic Review and Meta-Analysis. Front. Pharmacol..

[48] Hortobagyi G.N., Stemmer S.M., Burris H.A., Yap Y.-S., Sonke G.S., Paluch-Shimon S., Campone M., Blackwell K.L., André F., Winer E.P. (2016). Ribociclib as First-Line Therapy for HR-Positive, Advanced Breast Cancer. N. Engl. J. Med..

[49] Goetz M.P., Toi M., Campone M., Sohn J., Paluch-Shimon S., Huober J., Park I.H., Trédan O., Chen S.-C., Manso L. (2017). MONARCH 3: Abemaciclib As Initial Therapy for Advanced Breast Cancer. J. Clin. Oncol..

[50] Nanda R., Liu M.C., Yau C., Shatsky R., Pusztai L., Wallace A., Chien A.J., Forero-Torres A., Ellis E., Han H. (2020). Effect of Pembrolizumab Plus Neoadjuvant Chemotherapy on Pathologic Complete Response in Women With Early-Stage Breast Cancer: An Analysis of the Ongoing Phase 2 Adaptively Randomized I-SPY2 Trial. JAMA Oncol..

[51] Cardoso F., O’Shaughnessy J., Liu Z., McArthur H., Schmid P., Cortes J., Harbeck N., Telli M.L., Cescon D.W., Fasching P.A. (2025). Pembrolizumab and Chemotherapy in High-Risk, Early-Stage, ER+/HER2− Breast Cancer: A Randomized Phase 3 Trial. Nat. Med..

[52] Loi S., Salgado R., Curigliano G., Romero Díaz R.I., Delaloge S., Rojas García C.I., Kok M., Saura C., Harbeck N., Mittendorf E.A. (2025). Neoadjuvant Nivolumab and Chemotherapy in Early Estrogen Receptor-Positive Breast Cancer: A Randomized Phase 3 Trial. Nat. Med..

[53] Giuliano A.E., Ballman K.V., McCall L., Beitsch P.D., Brennan M.B., Kelemen P.R., Ollila D.W., Hansen N.M., Whitworth P.W., Blumencranz P.W. (2017). Effect of Axillary Dissection vs No Axillary Dissection on 10-Year Overall Survival Among Women With Invasive Breast Cancer and Sentinel Node Metastasis: The ACOSOG Z0011 (Alliance) Randomized Clinical Trial. JAMA.

[54] de Boniface J., Tvedskov T.F., Rydén L., Szulkin R., Reimer T., Kühn T., Kontos M., Gentilini O.D., Bagge R.O., Sund M. (2024). Omitting Axillary Dissection in Breast Cancer with Sentinel-Node Metastases. N. Engl. J. Med..

[55] Donker M., van Tienhoven G., Straver M.E., Meijnen P., van de Velde C.J.H., Mansel R.E., Cataliotti L., Westenberg A.H., Klinkenbijl J.H.G., Orzalesi L. (2014). Radiotherapy or Surgery of the Axilla after a Positive Sentinel Node in Breast Cancer (EORTC 10981-22023 AMAROS): A Randomised, Multicentre, Open-Label, Phase 3 Non-Inferiority Trial. Lancet Oncol..

[56] Geyer C.E., Garber J.E., Gelber R.D., Yothers G., Taboada M., Ross L., Rastogi P., Cui K., Arahmani A., Aktan G. (2022). Overall Survival in the OlympiA Phase III Trial of Adjuvant Olaparib in Patients with Germline Pathogenic Variants in *BRCA1/2* and High-Risk, Early Breast Cancer. Ann. Oncol..

[57] Johnston S.R.D., Harbeck N., Hegg R., Toi M., Martin M., Shao Z.M., Zhang Q.Y., Martinez Rodriguez J.L., Campone M., Hamilton E. (2020). Abemaciclib Combined With Endocrine Therapy for the Adjuvant Treatment of HR+, HER2−, Node-Positive, High-Risk, Early Breast Cancer (monarchE). J. Clin. Oncol..

[58] Slamon D., Lipatov O., Nowecki Z., McAndrew N., Kukielka-Budny B., Stroyakovskiy D., Yardley D.A., Huang C.-S., Fasching P.A., Crown J. (2024). Ribociclib plus Endocrine Therapy in Early Breast Cancer. N. Engl. J. Med..

[59] Weber W.P., Matrai Z., Hayoz S., Tausch C., Henke G., Zimmermann F., Montagna G., Fitzal F., Gnant M., Ruhstaller T. (2024). Tailored Axillary Surgery with or without Axillary Lymph Node Dissection Followed by Radiotherapy in Patients with Clinically Node-Positive Breast Cancer (OPBC-03/SAKK 23/16/IBCSG 57-18/ABCSG-53/GBG-101-TAXIS). J. Clin. Oncol..

[60] Park K.U., Somerfield M.R., Anne N., Brackstone M., Conlin A.K., Couto H.L., Dengel L.T., Eisen A., Harvey B.E., Hawley J. (2025). Sentinel Lymph Node Biopsy in Early-Stage Breast Cancer: ASCO Guideline Update. J. Clin. Oncol..

[61] Cao J.Q., Surgeoner B., Manna M., Boileau J.-F., Gelmon K.A., Brackstone M., Brezden-Masley C., Jerzak K.J., Prakash I., Sehdev S. (2024). Guidance for Canadian Breast Cancer Practice: National Consensus Recommendations for Clinical Staging of Patients Newly Diagnosed with Breast Cancer. Curr. Oncol..

[62] Gandhi S., Brackstone M., Hong N.J.L., Grenier D., Donovan E., Lu F.-I., Skarpathiotakis M., Lee J., Boileau J.-F., Perera F. (2022). A Canadian National Guideline on the Neoadjuvant Treatment of Invasive Breast Cancer, Including Patient Assessment, Systemic Therapy, and Local Management Principles. Breast Cancer Res. Treat..

[63] Boughey J., Suman V., Hunt K.J., Haffty B.G., Buchholz T., Symmans W.F., Rieken T.L., Dockter T.J., Campbell J.D., Weiss A. (2025). Abstract RF2-01: Factors Influencing Additional Nodal Disease and Pathologic Nodal Upstaging with Axillary Dissection in Patients with Residual Node-Positive Breast Cancer After Neoadjuvant Chemotherapy Enrolled on Alliance A011202 Clinical Trial. Clin. Cancer Res..

[64] Mamounas E.P., Bandos H., White J.R., Julian T.B., Khan A.J., Shaitelman S.F., Torres M.A., Vicini F.A., Ganz P.A., McCloskey S.A. (2025). Omitting Regional Nodal Irradiation after Response to Neoadjuvant Chemotherapy. N. Engl. J. Med..

[65] Jimenez R.B., Abdou Y., Anderson P., Barry P., Bradfield L., Bradley J.A., Heras L.D., Khan A., Matsen C., Rabinovitch R. (2025). Postmastectomy Radiation Therapy: An ASTRO-ASCO-SSO Clinical Practice Guideline. J. Clin. Oncol..

[66] Hills R., Bradley C., Braybrooke J., Davies L., Dodwell D., Mannu G., McGale P., Clarke M., Pan H., Berry R. (2025). Abstract LB1-01: Immediate Breast Surgery versus Deferral of Surgery in Women Aged 70+ Years with Operable Breast Cancer: Patient-Level Meta-Analysis of the Three Randomised Trials among 1082 Women. Clin. Cancer Res..

[67] Choosing Wisely, ABIM Foundation Society of Surgical Oncology Don’t Routinely Use Sentinel Node Biopsy in Clinically Node Negative Women ≥70 Years of Age with Early Stage Hormone Receptor Positive, HER2 Negative Invasive Breast Cancer. https://www.surgonc.org/wp-content/uploads/2024/04/FINAL_Choosing_Wisely_5.30.23-SSO-5things-List_2021-Updates.pdf.

[68] Ditsch N., Gnant M., Thomssen C., Harbeck N. (2025). St. Gallen/Vienna 2025 Summary of Key Messages on Therapy in Early Breast Cancer from the 2025 St. Gallen International Breast Cancer Conference. Breast Care.

[69] Esserman L.J., Yau C., Thompson C.K., van ’t Veer L.J., Borowsky A.D., Hoadley K.A., Tobin N.P., Nordenskjöld B., Fornander T., Stål O. (2017). Use of Molecular Tools to Identify Patients With Indolent Breast Cancers With Ultralow Risk Over 2 Decades. JAMA Oncol..

[70] Denduluri N., Somerfield M.R., Eisen A., Holloway J.N., Hurria A., King T.A., Lyman G.H., Partridge A.H., Telli M.L., Trudeau M.E. (2016). Selection of Optimal Adjuvant Chemotherapy Regimens for Human Epidermal Growth Factor Receptor 2 (HER2)–Negative and Adjuvant Targeted Therapy for HER2-Positive Breast Cancers: An American Society of Clinical Oncology Guideline Adaptation of the Cancer Care Ontario Clinical Practice Guideline. J. Clin. Oncol..

[71] Denduluri N., Chavez-MacGregor M., Telli M.L., Eisen A., Graff S.L., Hassett M.J., Holloway J.N., Hurria A., King T.A., Lyman G.H. (2018). Selection of Optimal Adjuvant Chemotherapy and Targeted Therapy for Early Breast Cancer: ASCO Clinical Practice Guideline Focused Update. J. Clin. Oncol..

[72] Denduluri N., Somerfield M.R., Chavez-MacGregor M., Comander A.H., Dayao Z., Eisen A., Freedman R.A., Gopalakrishnan R., Graff S.L., Hassett M.J. (2020). Selection of Optimal Adjuvant Chemotherapy and Targeted Therapy for Early Breast Cancer: ASCO Guideline Update. J. Clin. Oncol..

[73] Nitz U., Gluz O., Clemens M., Malter W., Reimer T., Nuding B., Aktas B., Stefek A., Pollmanns A., Lorenz-Salehi F. (2019). West German Study PlanB Trial: Adjuvant Four Cycles of Epirubicin and Cyclophosphamide Plus Docetaxel Versus Six Cycles of Docetaxel and Cyclophosphamide in HER2-Negative Early Breast Cancer. J. Clin. Oncol..

[74] De Gregorio A., Janni W., Friedl T.W.P., Nitz U., Rack B., Schneeweiss A., Kates R., Fehm T., Kreipe H., Christgen M. (2022). The Impact of Anthracyclines in Intermediate and High-Risk HER2-Negative Early Breast Cancer—A Pooled Analysis of the Randomised Clinical Trials PlanB and SUCCESS C. Br. J. Cancer.

[75] Caparica R., Bruzzone M., Poggio F., Ceppi M., De Azambuja E., Lambertini M. (2019). Anthracycline and Taxane-Based Chemotherapy versus Docetaxel and Cyclophosphamide in the Adjuvant Treatment of HER2-Negative Breast Cancer Patients: A Systematic Review and Meta-Analysis of Randomized Controlled Trials. Breast Cancer Res. Treat..

[76] Giffoni De Mello Morais Mata D., Rush M.-B., Smith-Uffen M., Younus J., Lohmann A.E., Trudeau M., Morgan R.L. (2024). The Omission of Anthracycline Chemotherapy in Women with Early HER2-Negative Breast Cancer—A Systematic Review and Meta-Analysis. Curr. Oncol..

[77] Blum J.L., Flynn P.J., Yothers G., Asmar L., Geyer C.E., Jacobs S.A., Robert N.J., Hopkins J.O., O’Shaughnessy J.A., Dang C.T. (2017). Anthracyclines in Early Breast Cancer: The ABC Trials—USOR 06-090, NSABP B-46-I/USOR 07132, and NSABP B-49 (NRG Oncology). J. Clin. Oncol..

[78] Basu A., Dabak V.S., Loutfi R. (2018). Four versus Six Cycles of Docetaxel and Cyclophosphamide (TC) in Early Stage Hormone Positive Breast Cancer. J. Clin. Oncol..

[79] Chen N., Freeman J.Q., Yarlagadda S., Atmakuri A., Kalinksy K., Pusztai L., Huo D., Nanda R., Howard F. (2024). Impact of Anthracyclines in High Genomic Risk Node-Negative HR+/HER2− Breast Cancer. San Antonio Breast Cancer Symp. SABCS.

[80] O’Shaughnessy J., Graham C.L., Whitworth P., Beitsch P.D., Osborne C.R.C., Layeequr Rahman R., Brown E.A., Gold L.P., Johnson N.M., Brufsky A. (2024). Association of MammaPrint Index and 3-Year Outcome of Patients with HR+HER2− Early-Stage Breast Cancer Treated with Chemotherapy with or without Anthracycline. J. Clin. Oncol..

[81] Armenian S.H., Lacchetti C., Barac A., Carver J., Constine L.S., Denduluri N., Dent S., Douglas P.S., Durand J.-B., Ewer M. (2017). Prevention and Monitoring of Cardiac Dysfunction in Survivors of Adult Cancers: American Society of Clinical Oncology Clinical Practice Guideline. J. Clin. Oncol..

[82] Ewer M.S., Ewer S.M. (2015). Cardiotoxicity of Anticancer Treatments. Nat. Rev. Cardiol..

[83] Swain S.M., Whaley F.S., Ewer M.S. (2003). Congestive Heart Failure in Patients Treated with Doxorubicin: A Retrospective Analysis of Three Trials. Cancer.

[84] Abdallah K., Majeed Z., Moudgil R. (2024). Breast Cancer Therapies: A Cardiac Perspective. JCO Oncol. Pract..

[85] Cardinale D., Colombo A., Bacchiani G., Tedeschi I., Meroni C.A., Veglia F., Civelli M., Lamantia G., Colombo N., Curigliano G. (2015). Early Detection of Anthracycline Cardiotoxicity and Improvement with Heart Failure Therapy. Circulation.

[86] Zagami P., Trapani D., Nicolò E., Corti C., Valenza C., Criscitiello C., Curigliano G., Carey L.A. (2024). Cardiotoxicity of Agents Used in Patients With Breast Cancer. JCO Oncol. Pract..

[87] Thavendiranathan P., Abdel-Qadir H., Fischer H.D., Camacho X., Amir E., Austin P.C., Lee D.S. (2016). Breast Cancer Therapy-Related Cardiac Dysfunction in Adult Women Treated in Routine Clinical Practice: A Population-Based Cohort Study. J. Clin. Oncol..

[88] Vo J.B., Ramin C., Veiga L.H.S., Brandt C., Curtis R.E., Bodelon C., Barac A., Roger V.L., Feigelson H.S., Buist D.S.M. (2024). Long-Term Cardiovascular Disease Risk after Anthracycline and Trastuzumab Treatments in US Breast Cancer Survivors. J. Natl. Cancer Inst..

[89] Ling G., Ge F., Li W., Wei Y., Guo S., Zhang Y., Li Y., Zhang Y., Liu H., Wu Y. (2025). Anthracycline-Induced Cardiotoxicity: Emerging Mechanisms and Therapies. Med. Plus.

[90] Armenian S.H., Xu L., Ky B., Sun C., Farol L.T., Pal S.K., Douglas P.S., Bhatia S., Chao C. (2016). Cardiovascular Disease Among Survivors of Adult-Onset Cancer: A Community-Based Retrospective Cohort Study. J. Clin. Oncol..

[91] Dent S., Guha A., Moore H., Makari D., McCaleb R., Arias I., Stergiopoulos S., Li B., Fradley M. (2025). CARDIAC-STAR: Prevalence of Cardiovascular Comorbidities in Patients with HR+/HER2− Metastatic Breast Cancer. Cardio-Oncology.

[92] Davies C., Godwin J., Gray R., Clarke M., Cutter D., Darby S., McGale P., Pan H.C., Taylor C., Early Breast Cancer Trialists’ Collaborative Group (EBCTCG) (2011). Relevance of Breast Cancer Hormone Receptors and Other Factors to the Efficacy of Adjuvant Tamoxifen: Patient-Level Meta-Analysis of Randomised Trials. Lancet.

[93] Francis P.A., Fleming G.F., Láng I., Ciruelos E.M., Bonnefoi H.R., Bellet M., Bernardo A., Climent M.A., Martino S., Bermejo B. (2023). Adjuvant Endocrine Therapy in Premenopausal Breast Cancer: 12-Year Results From SOFT. J. Clin. Oncol..

[94] Davies C., Pan H., Godwin J., Gray R., Arriagada R., Raina V., Abraham M., Medeiros Alencar V.H., Badran A., Bonfill X. (2013). Long-Term Effects of Continuing Adjuvant Tamoxifen to 10 Years versus Stopping at 5 Years after Diagnosis of Oestrogen Receptor-Positive Breast Cancer: ATLAS, a Randomised Trial. Lancet.

[95] Gray R.G., Rea D., Handley K., Bowden S.J., Perry P., Earl H.M., Poole C.J., Bates T., Chetiyawardana S., Dewar J.A. (2013). aTTom: Long-Term Effects of Continuing Adjuvant Tamoxifen to 10 Years versus Stopping at 5 Years in 6953 Women with Early Breast Cancer. J. Clin. Oncol..

[96] Burstein H.J., Lacchetti C., Anderson H., Buchholz T.A., Davidson N.E., Gelmon K.A., Giordano S.H., Hudis C.A., Solky A.J., Stearns V. (2019). Adjuvant Endocrine Therapy for Women With Hormone Receptor–Positive Breast Cancer: ASCO Clinical Practice Guideline Focused Update. J. Clin. Oncol..

[97] Pistilli B., Lohrisch C., Sheade J., Fleming G.F. (2022). Personalizing Adjuvant Endocrine Therapy for Early-Stage Hormone Receptor–Positive Breast Cancer. Am. Soc. Clin. Oncol. Educ. Book.

[98] Freedman R.A., Caswell-Jin J.L., Hassett M., Somerfield M.R., Giordano S.H., Chavez-MacGregor M., Comander A.H., Dayao Z., Eisen A., For the Optimal Adjuvant Chemotherapy and Targeted Therapy for Early Breast Cancer Guideline Expert Panel (2024). Optimal Adjuvant Chemotherapy and Targeted Therapy for Early Breast Cancer—Cyclin-Dependent Kinase 4 and 6 Inhibitors: ASCO Guideline Rapid Recommendation Update. J. Clin. Oncol..

[99] Pan H., Gray R., Braybrooke J., Davies C., Taylor C., McGale P., Peto R., Pritchard K.I., Bergh J., Dowsett M. (2017). 20-Year Risks of Breast-Cancer Recurrence after Stopping Endocrine Therapy at 5 Years. N. Engl. J. Med..

[100] Rastogi P., O’Shaughnessy J., Martin M., Boyle F., Cortes J., Rugo H.S., Goetz M.P., Hamilton E.P., Huang C.-S., Senkus E. (2024). Adjuvant Abemaciclib Plus Endocrine Therapy for Hormone Receptor–Positive, Human Epidermal Growth Factor Receptor 2–Negative, High-Risk Early Breast Cancer: Results From a Preplanned monarchE Overall Survival Interim Analysis, Including 5-Year Efficacy Outcomes. J. Clin. Oncol..

[101] Breast Cancer Canada 2024 Progress Report. https://www.flipsnack.com/E7FBBD99E8C/2024-progress-report/full-view.html.

[102] Johnston S., Martin M., O’Shaughnessy J., Hegg R., Tolaney S.M., Guarneri V., Mastro L.D., Campone M., Sohn J., Boyle F. (2025). Overall Survival with Abemaciclib in Early Breast Cancer. Ann. Oncol..

[103] Hortobagyi G.N., Lacko A., Sohn J., Cruz F., Ruiz Borrego M., Manikhas A., Hee Park Y., Stroyakovskiy D., Yardley D.A., Huang C.-S. (2025). A Phase III Trial of Adjuvant Ribociclib plus Endocrine Therapy versus Endocrine Therapy Alone in Patients with HR-Positive/HER2-Negative Early Breast Cancer: Final Invasive Disease-Free Survival Results from the NATALEE Trial. Ann. Oncol..

[104] Fasching P.A., Stroyakovskiy D., Yardley D.A., Huang C.-S., Crown J., Bardia A., Chia S., Im S.-A., Martin M., Xu B. (2025). Ribociclib Plus Endocrine Therapy in Hormone Receptor–Positive/*ERBB2* -Negative Early Breast Cancer: 4-Year Outcomes from the NATALEE Randomized Clinical Trial. JAMA Oncol..

[105] Crown J., Stroyakovskii D., Yardley D.A., Huang C.-S., Fasching P.A., Bardia A., Chia S., Im S.-A., Martin M., Xu B. (2025). Adjuvant Ribociclib plus Nonsteroidal Aromatase Inhibitor Therapy in Patients with HR-Positive/HER2-Negative Early Breast Cancer: 5-Year Follow-up of NATALEE Efficacy Outcomes and Updated Overall Survival. ESMO Open.

[106] Hussain M., Brezden-Masley C., Chia S., Curigliano G., Webster M., Henning J.-W. (2025). Clinician’s Guide: Expert Insights on the Use of CDK4/6 Inhibitors in Patients with Early Breast Cancer. Ther. Adv. Med. Oncol..

[107] Jerzak K.J., Sehdev S., Boileau J.-F., Brezden-Masley C., Califaretti N., Edwards S., Gordon J., Henning J.-W., LeVasseur N., Railton C. (2025). Multidisciplinary Practical Guidance for Implementing Adjuvant CDK4/6 Inhibitors for Patients with HR-Positive, HER2-Negative Early Breast Cancer in Canada. Curr. Oncol..

[108] Gray R., Bradley R., Braybrooke J., Clarke M., Hills R.K., Peto R., Bergh J.C.S., Swain S.M., Davidson N.E., Francis P.A. (2023). Effects of Ovarian Ablation or Suppression on Breast Cancer Recurrence and Survival: Patient-Level Meta-Analysis of 14,993 Pre-Menopausal Women in 25 Randomized Trials. J. Clin. Oncol..

[109] Pagani O., Walley B.A., Fleming G.F., Colleoni M., Láng I., Gomez H.L., Tondini C., Burstein H.J., Goetz M.P., Ciruelos E.M. (2023). Adjuvant Exemestane With Ovarian Suppression in Premenopausal Breast Cancer: Long-Term Follow-Up of the Combined TEXT and SOFT Trials. J. Clin. Oncol..

[110] Bradley R., Braybrooke J., Gray R., Hills R.K., Liu Z., Pan H., Peto R., Dodwell D., McGale P., Taylor C. (2022). Aromatase Inhibitors versus Tamoxifen in Premenopausal Women with Oestrogen Receptor-Positive Early-Stage Breast Cancer Treated with Ovarian Suppression: A Patient-Level Meta-Analysis of 7030 Women from Four Randomised Trials. Lancet Oncol..

[111] (2024). Novartis Pharmaceuticals A Phase IIIb Study to Characterize the Effectiveness and Safety of Adjuvant Ribociclib in a Wide Patient Population with HR+ HER2− Early Breast Cancer (Adjuvant WIDER).

[112] Early Breast Cancer Trialists’ Collaborative Group (EBCTCG) (2015). Aromatase Inhibitors versus Tamoxifen in Early Breast Cancer: Patient-Level Meta-Analysis of the Randomised Trials. Lancet.

[113] (2009). The BIG 1-98 Collaborative Group Letrozole Therapy Alone or in Sequence with Tamoxifen in Women with Breast Cancer. N. Engl. J. Med..

[114] Zhang Y., Schnabel C.A., Schroeder B.E., Jerevall P.-L., Jankowitz R.C., Fornander T., Stål O., Brufsky A.M., Sgroi D., Erlander M.G. (2013). Breast Cancer Index Identifies Early-Stage Estrogen Receptor–Positive Breast Cancer Patients at Risk for Early- and Late-Distant Recurrence. Clin. Cancer Res..

[115] Sparano J.A., Crager M., Gray R.J., Tang G., Hoag J., Baehner F.L., Shak S., Makower D.F., Albain K.S., Hayes D.F. (2024). Clinical and Genomic Risk for Late Breast Cancer Recurrence and Survival. NEJM Evid..

[116] Gomis R.R., Gawrzak S. (2017). Tumor Cell Dormancy. Mol. Oncol..

[117] Pedersen R.N., Esen B.Ö., Mellemkjær L., Christiansen P., Ejlertsen B., Lash T.L., Nørgaard M., Cronin-Fenton D. (2022). The Incidence of Breast Cancer Recurrence 10–32 Years After Primary Diagnosis. J. Natl. Cancer Inst..

[118] Goss P.E., Ingle J.N., Martino S., Robert N.J., Muss H.B., Piccart M.J., Castiglione M., Tu D., Shepherd L.E., Pritchard K.I. (2005). Randomized Trial of Letrozole Following Tamoxifen as Extended Adjuvant Therapy in Receptor-Positive Breast Cancer: Updated Findings from NCIC CTG MA.17. J. Natl. Cancer Inst..

[119] Goss P.E., Ingle J.N., Pritchard K.I., Robert N.J., Muss H., Gralow J., Gelmon K., Whelan T., Strasser-Weippl K., Rubin S. (2016). Extending Aromatase-Inhibitor Adjuvant Therapy to 10 Years. N. Engl. J. Med..

[120] Del Mastro L., Mansutti M., Bisagni G., Ponzone R., Durando A., Amaducci L., Campadelli E., Cognetti F., Frassoldati A., Michelotti A. (2021). Extended Therapy with Letrozole as Adjuvant Treatment of Postmenopausal Patients with Early-Stage Breast Cancer: A Multicentre, Open-Label, Randomised, Phase 3 Trial. Lancet Oncol..

[121] Gnant M., Fitzal F., Rinnerthaler G., Steger G.G., Greil-Ressler S., Balic M., Heck D., Jakesz R., Thaler J., Egle D. (2021). Duration of Adjuvant Aromatase-Inhibitor Therapy in Postmenopausal Breast Cancer. N. Engl. J. Med..

[122] Braybrooke J., Bradley R., Hills R.K., Peto R., Kerr A., Pan H., Clarke M., Dodwell D., McGale P., Taylor C. (2025). Extending the Duration of Endocrine Treatment for Early Breast Cancer: Patient-Level Meta-Analysis of 12 Randomised Trials of Aromatase Inhibitors in 22 031 Postmenopausal Women Already Treated with at Least 5 Years of Endocrine Therapy. Lancet.

[123] Bekes I., Huober J. (2023). Extended Adjuvant Endocrine Therapy in Early Breast Cancer Patients—Review and Perspectives. Cancers.

[124] O’Shaughnessy J., Tolaney S.M., Yardley D.A., Hart L., Razavi P., Fasching P.A., Janni W., Schwartzberg L., Kim J., Akdere M. (2025). Real-World Risk of Recurrence and Treatment Outcomes with Adjuvant Endocrine Therapy in Patients with Stage II-III HR+/HER2− Early Breast Cancer. Breast.

[125] Jhaveri K., Pegram M., Neven P., Curigliano G., Spring L.M., Gligorov J., Schlam I., Harbeck N., Juric D., Lim E. (2024). 292P Real-World Evidence on Risk of Recurrence (ROR) in Patients (Pts) with Node-Negative (N0) and Node-Positive HR+/HER2– Early Breast Cancer (EBC) from US Electronic Health Records (EHR). Ann. Oncol..

[126] Conley C.C., McIntyre M., Pensak N.A., Lynce F., Graham D., Ismail-Khan R., Lopez K., Vadaparampil S.T., O’Neill S.C. (2022). Barriers and Facilitators to Taking CDK4/6 Inhibitors among Patients with Metastatic Breast Cancer: A Qualitative Study. Breast Cancer Res. Treat..

[127] Welslau M.K., Fasching P., Mueller L., Belleville E., Rieger L., Zahn M.-O., Lex B., Häberle L., Tesch H. (2023). 378O Persistence under Abemaciclib and Endocrine Treatment (ABA+ET) in Patients with Advanced Breast Cancer (aBC): First Results of the Randomized IMPACT Trial Comparing Patient Coaching with the MASCC Oral Agent Teaching Tool (MOATT) versus Local Routine Patient Coaching (LC). Ann. Oncol..

[128] Mayer E.L., Trapani D., Kim S.-E., Faggen M., Sinclair N., Sanz-Altamira P., Battelli C., Berwick S., Lo S., Acevedo J. (2025). TRADE: A Phase II Trial to Assess the Tolerability of Abemaciclib Dose Escalation in Early-Stage HR+/HER2− Breast Cancer. Ann. Oncol..

[129] Chan A., Nixon N., Al-Khaifi M., Bestavros A., Blyth C., Cheung W.Y., Hamm C., Joly-Mischlich T., Manna M., McFarlane T. (2025). Optimizing Adjuvant Care in Early Breast Cancer: Multidisciplinary Strategies and Innovative Models from Canadian Centers. Curr. Oncol..

[130] Bedrosian I., Somerfield M.R., Achatz M.I., Boughey J.C., Curigliano G., Friedman S., Kohlmann W.K., Kurian A.W., Laronga C., Lynce F. (2024). Germline Testing in Patients With Breast Cancer: ASCO-Society of Surgical Oncology Guideline. J. Clin. Oncol..

[131] Weber E., Carmona-Gonzalez C.A., Boucher M., Eisen A., Laing K., Melvin J., Schrader K.A., Sehdev S., Wong S.M., Gelmon K.A. (2025). Canadian Recommendations for Germline Genetic Testing of Patients with Breast Cancer: A Call to Action. Curr. Oncol..

[132] Choong G.M., Hoskin T.L., Boughey J.C., Ingle J.N., Goetz M.P. (2025). Endocrine Therapy Omission in Estrogen Receptor–Low (1%–10%) Early-Stage Breast Cancer. J. Clin. Oncol..

[133] Schmid P., Cortes J., Dent R., Pusztai L., McArthur H., Kümmel S., Bergh J., Denkert C., Park Y.H., Hui R. (2022). Event-Free Survival with Pembrolizumab in Early Triple-Negative Breast Cancer. N. Engl. J. Med..

[134] Early Breast Cancer Trialists’ Collaborative Group (EBCTCG) (2015). Adjuvant Bisphosphonate Treatment in Early Breast Cancer: Meta-Analyses of Individual Patient Data from Randomised Trials. Lancet.

[135] Gralow J.R., Barlow W.E., Paterson A.H.G., M’iao J.L., Lew D.L., Stopeck A.T., Hayes D.F., Hershman D.L., Schubert M.M., Clemons M. (2020). Phase III Randomized Trial of Bisphosphonates as Adjuvant Therapy in Breast Cancer: S0307. J. Natl. Cancer Inst..

[136] Eisen A., Somerfield M.R., Accordino M.K., Blanchette P.S., Clemons M.J., Dhesy-Thind S., Dillmon M.S., D’Oronzo S., Fletcher G.G., Frank E.S. (2022). Use of Adjuvant Bisphosphonates and Other Bone-Modifying Agents in Breast Cancer: ASCO-OH (CCO) Guideline Update. J. Clin. Oncol..

[137] Coleman R., Hadji P., Body J.-J., Santini D., Chow E., Terpos E., Oudard S., Bruland Ø., Flamen P., Kurth A. (2020). Bone Health in Cancer: ESMO Clinical Practice Guidelines. Ann. Oncol..

[138] Friedl T.W.P., Fehm T., Müller V., Lichtenegger W., Blohmer J., Lorenz R., Forstbauer H., Fink V., Bekes I., Huober J. (2021). Prognosis of Patients With Early Breast Cancer Receiving 5 Years vs 2 Years of Adjuvant Bisphosphonate Treatment: A Phase 3 Randomized Clinical Trial. JAMA Oncol..

[139] Mittal A., Tamimi F., Molto C., Di Iorio M., Amir E. (2024). Benefit of Adjuvant Bisphosphonates in Early Breast Cancer Treated with Contemporary Systemic Therapy: A Meta-Analysis of Randomized Control Trials. Heliyon.

[140] Desnoyers A., Amir E., Tannock I.F. (2021). Adjuvant Zoledronate Therapy for Women With Breast Cancer—Effective Treatment or Fool’s Gold?. JAMA Oncol..

[141] Jacobs C., Amir E., Paterson A., Zhu X., Clemons M. (2015). Are Adjuvant Bisphosphonates Now Standard of Care of Women with Early Stage Breast Cancer? A Debate from the Canadian Bone and the Oncologist New Updates Meeting. J. Bone Oncol..

[142] Gnant M., Pfeiler G., Steger G.G., Egle D., Greil R., Fitzal F., Wette V., Balic M., Haslbauer F., Melbinger-Zeinitzer E. (2019). Adjuvant Denosumab in Postmenopausal Patients with Hormone Receptor-Positive Breast Cancer (ABCSG-18): Disease-Free Survival Results from a Randomised, Double-Blind, Placebo-Controlled, Phase 3 Trial. Lancet Oncol..

[143] Coleman R., Finkelstein D.M., Barrios C., Martin M., Iwata H., Hegg R., Glaspy J., Periañez A.M., Tonkin K., Deleu I. (2020). Adjuvant Denosumab in Early Breast Cancer (D-CARE): An International, Multicentre, Randomised, Controlled, Phase 3 Trial. Lancet Oncol..

[144] Jerzak K.J., Raphael J., Desautels D., Blanchette P.S., Tyono I., Pritchard K.I. Bone-Targeted Therapy in Early Breast Cancer|CancerNetwork. https://www.cancernetwork.com/view/bone-targeted-tx.

[145] Coleman R., Cameron D., Dodwell D., Bell R., Wilson C., Rathbone E., Keane M., Gil M., Burkinshaw R., Grieve R. (2014). Adjuvant Zoledronic Acid in Patients with Early Breast Cancer: Final Efficacy Analysis of the AZURE (BIG 01/04) Randomised Open-Label Phase 3 Trial. Lancet Oncol..

[146] Su H.I., Lacchetti C., Letourneau J., Partridge A.H., Qamar R., Quinn G.P., Reinecke J., Smith J.F., Tesch M., Wallace W.H. (2025). Fertility Preservation in People With Cancer: ASCO Guideline Update. J. Clin. Oncol..

[147] Peccatori F.A., Azim H.A., Orecchia R., Hoekstra H.J., Pavlidis N., Kesic V., Pentheroudakis G. (2013). Cancer, Pregnancy and Fertility: ESMO Clinical Practice Guidelines for Diagnosis, Treatment and Follow-Up^†^. Ann. Oncol..

[148] Sorouri K., Loren A.W., Amant F., Partridge A.H. (2023). Patient-Centered Care in the Management of Cancer During Pregnancy. Am. Soc. Clin. Oncol. Educ. Book.

[149] Roesch E., Maggiotto A., Valente S.A. (2025). Multidisciplinary Management of Pregnancy-Associated Breast Cancer. JCO Oncol. Pract..

[150] Kesireddy M., Krishnamurthy J. (2025). Pregnancy-Associated Breast Cancer: Key Concepts for Optimizing Diagnosis and Treatment. JCO Oncol. Pract..

[151] Peccatori F.A., Azim H.A., Scarfone G., Gadducci A., Bonazzi C., Gentilini O., Galimberti V., Intra M., Locatelli M., Acaia B. (2009). Weekly Epirubicin in the Treatment of Gestational Breast Cancer (GBC). Breast Cancer Res. Treat..

[152] García-Manero M., Royo M.P., Espinos J., Pina L., Alcazar J.L., López G. (2009). Pregnancy Associated Breast Cancer. Eur. J. Surg. Oncol..

[153] Cardonick E., Dougherty R., Grana G., Gilmandyar D., Ghaffar S., Usmani A. (2010). Breast Cancer During Pregnancy: Maternal and Fetal Outcomes. Cancer J..

[154] Loibl S., Han S.N., von Minckwitz G., Bontenbal M., Ring A., Giermek J., Fehm T., Calsteren K.V., Linn S.C., Schlehe B. (2012). Treatment of Breast Cancer during Pregnancy: An Observational Study. Lancet Oncol..

[155] Murthy R.K., Theriault R.L., Barnett C.M., Hodge S., Ramirez M.M., Milbourne A., Rimes S.A., Hortobagyi G.N., Valero V., Litton J.K. (2014). Outcomes of Children Exposed in Uteroto Chemotherapy for Breast Cancer. Breast Cancer Res..

[156] Safi N., Anazodo A., Dickinson J.E., Lui K., Wang A.Y., Li Z., Sullivan E.A. (2019). In Utero Exposure to Breast Cancer Treatment: A Population-Based Perinatal Outcome Study. Br. J. Cancer.

[157] O’Laughlin A., So S., Fleischer L., Akoto S., Cardonick E. (2019). Safety of Taxane Chemotherapy in Breast Cancer During Pregnancy [28O]. Obstet. Gynecol..

[158] Poggio F., Tagliamento M., Pirrone C., Soldato D., Conte B., Molinelli C., Cosso M., Fregatti P., Del Mastro L., Lambertini M. (2020). Update on the Management of Breast Cancer during Pregnancy. Cancers.

[159] Buonomo B., Brunello A., Noli S., Miglietta L., Del Mastro L., Lambertini M., Peccatori F.A. (2019). Tamoxifen Exposure during Pregnancy: A Systematic Review and Three More Cases. Breast Care.

[160] Partridge A.H., Niman S.M., Ruggeri M., Peccatori F.A., Azim H.A., Colleoni M., Saura C., Shimizu C., Sætersdal A.B., Kroep J.R. (2023). Interrupting Endocrine Therapy to Attempt Pregnancy after Breast Cancer. N. Engl. J. Med..

[161] Pagani O., Niman S.M., Ruggeri M., Peccatori F.A., Azim H.A., Colleoni M.A., Manich C.S., Shimizu C., Satersdal A., Kroep J.R. (2025). 5-Year Follow-up Results from the POSITIVE (Pregnancy Outcome and Safety of Interrupting Therapy for Women with Endocrine Responsive Breast Cancer) Trial. ESMO Open.

