# Supplementary material for: Guidance for Canadian Breast Cancer Practice: National Consensus Recommendations for the Systemic Treatment of Patients with HR+/HER2− Early Breast Cancer 2025
Source: Curr Oncol. 2026 Feb 12;33(2):112. doi: 10.3390/curroncol33020112 (PMC12939464; doi:10.3390/curroncol33020112)
Supplement: Supplementary file 1 [file curroncol-33-00112-s001.zip › curroncol-4059928-supplementary.pdf]

Voting Results

Guidance for Canadian Breast Cancer Practice: National Consensus Recommendations for the Systemic Treatment of Patients with HR+/HER2– Early Breast Cancer

Voting results (Agree with statement as is, Agree with statement with edits, Do not agree with statement, Abstain)

		Consensus Recommendation	Consensus was reached on:
1	For patients with HR+/HER2– EBC where chemotherapy is clearly indicated and surgical information will not alter that decision, NAC with shared decision-making should be considered. (Moderate Recommendation)	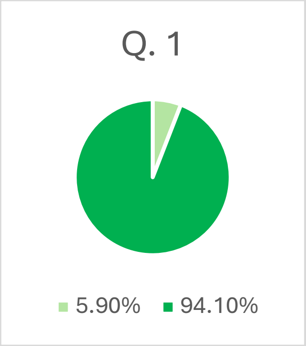<br>N = 17	2nd round
2	For patients with HR+/HER2– EBC in whom the benefit of chemotherapy is uncertain, and where NAC is being considered through shared decision-making, genomic recurrence risk testing on core biopsy should be considered to help guide treatment decisions. (Moderate Recommendation)	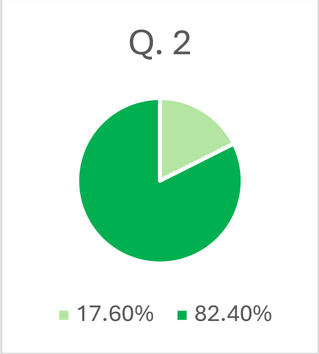<br>N = 17	2nd round

3. a)	a) For premenopausal patients with T1–2, biopsy-proven N+, HR+/HER2– EBC, NAC could be considered, especially in patients with high-risk features. (Weak Recommendation)	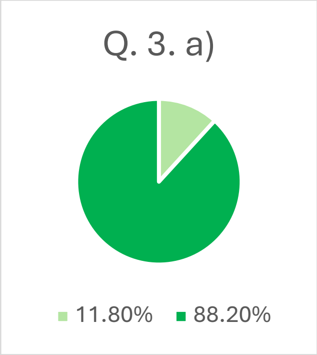 N=17	2nd round
3. b)	b) For premenopausal patients with non-metastatic, inoperable T3–4 disease, neoadjuvant systemic therapy is standard of care and can potentially render an inoperable patient operable. (Strong Recommendation)	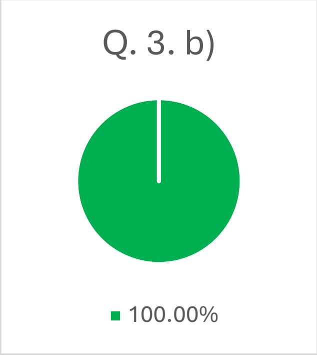 N=17	3rd round
4. a)	a) For postmenopausal patients with T1–2, biopsy-proven N1, HR+/HER2– EBC, upfront surgery is standard of care. (Strong recommendation) If neoadjuvant therapy is being considered, genomic recurrence risk testing should be used to aid decision-making. (Moderate recommendation)	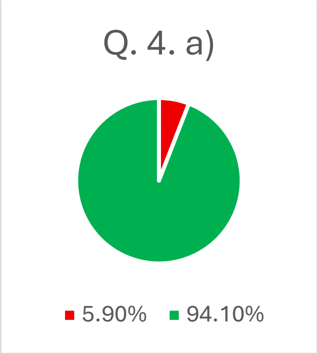 N=17	3rd round

4. b)	b) For postmenopausal patients with non-metastatic inoperable T3–4 disease, neoadjuvant systemic therapy is standard of care and can potentially render a patient with inoperable disease operable. (Strong recommendation)	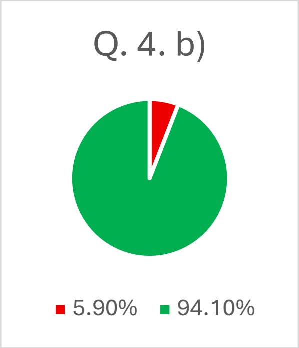<br>N=17	3rd round
5	For patients with cN2–3, HR+/HER2– EBC, NAC is the standard of care, given its potential to downstage nodal disease and improve surgical options. (Strong Recommendation)	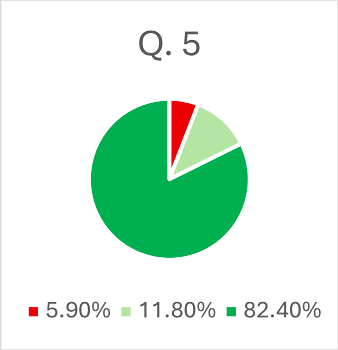<br>N=17	2nd round
6. a)	For patients with cN+, HR+/HER2– EBC where chemotherapy is indicated, a taxane-based regimen +/- anthracycline is recommended based on clinical risk, comorbidities, and shared-decision-making. (Strong Recommendation)<br><br>Although consensus was achieved on Round 2, the group continued to discuss and refine this statement to address the nuance in this area.	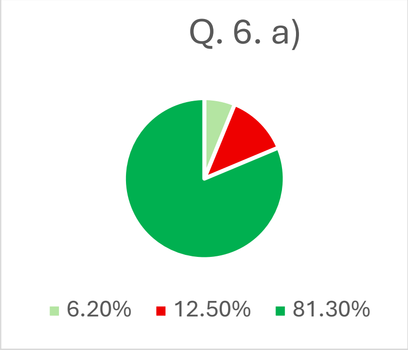<br>N = 17<br>1 abstained	2nd round

6. b)	For patients with cN+, HR+/HER2– EBC with cardiac or other contraindications to anthracyclines, concerns about long-term toxicity, and/or who decline anthracycline use after shared decision-making, a non-anthracycline-based regimen (e.g., docetaxel + cyclophosphamide) is standard of care. (Strong Recommendation)	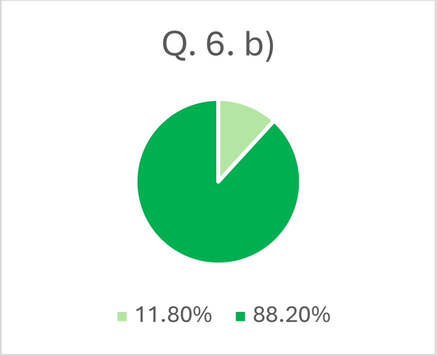<br>N = 17	3rd round
7.	For patients with inoperable, locally advanced or inflammatory HR+/HER2– EBC who are appropriate candidates for chemotherapy, NAC with an anthracycline-taxane-based regimen is standard of care. (Strong Recommendation)	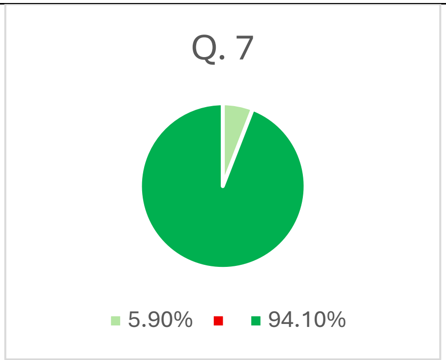<br>N=17	2nd round
8.	For patients with inoperable breast cancer (potentially operable with downstaging) where chemotherapy is inappropriate *or* contraindicated, NET is a standard of care with the goal of proceeding with surgery. (Strong Recommendation)	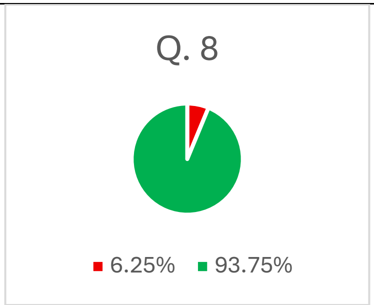<br>1 abstain<br>N=17	3rd round

9.	Routine use of neoadjuvant CDK4/6i + ET with curative intent is not recommended. (Strong Recommendation)	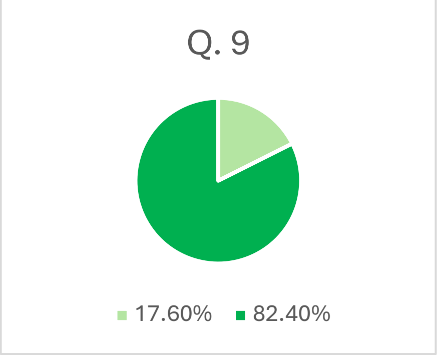 N=17	2nd round
10.	For patients with HR+/HER2– EBC, neoadjuvant immune checkpoint inhibition + chemotherapy is not currently recommended. This approach remains under active investigation. (Strong Recommendation)	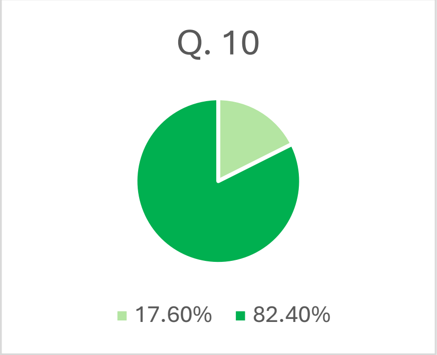 N=17	2nd round
11. a)	For patients with HR+/HER2– EBC and 1-2 positive sentinel nodes, performing further node dissection (e.g., ALND) to determine adjuvant systemic therapy is not recommended. (Strong Recommendation)	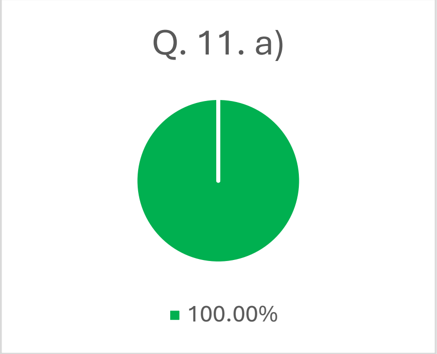	2nd round

11. b)	For patients with HR+/HER2– EBC and ⩾3 positive sentinel nodes, MDT discussion (where available) regarding loco-regional management is recommended. (Strong Recommendation)	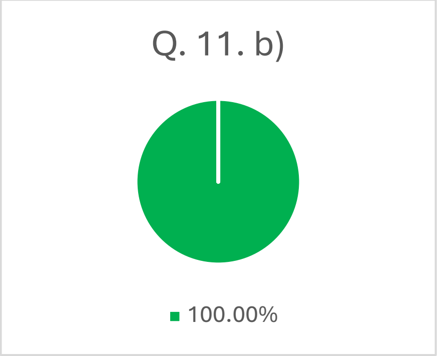<br>2 abstain<br>N=17	2nd round
12.	For patients with HR+/HER2– EBC where NAC is planned, placement of a clip to mark biopsied lesions in both breast and lymph nodes is standard of care to aid surgical planning. (Strong Recommendation)	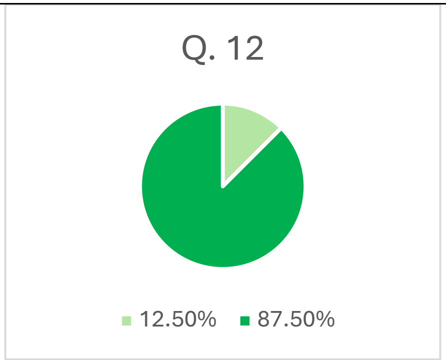<br>N=17	2nd round
13.	For patients with clinical Stage I-II HR+/HER2– EBC with low-risk features, upfront surgery is the standard of care, with adjuvant systemic therapy guided by final pathology. (Strong Recommendation)	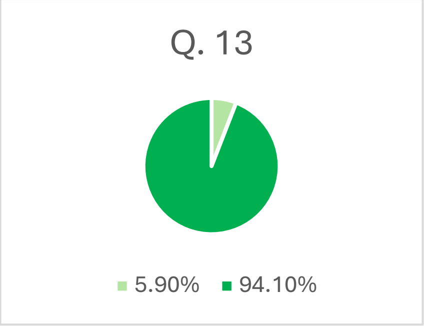<br>N=17	2nd round

14.	For patients aged ⩾70 years with operable HR+/HER2 – EBC who are fit for surgery, definitive surgery is standard of care. (Strong Recommendation)	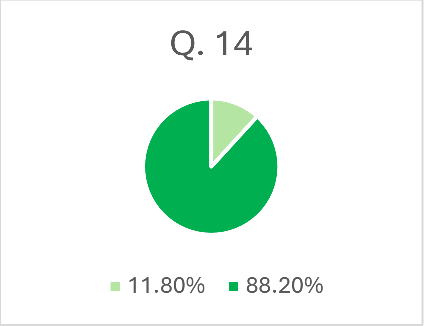<br>N=17	2nd round
15. a)	For patients with ≥T1bN0 HR+/HER2– EBC who are eligible for chemotherapy, and who have other intermediate- or high-risk features, genomic recurrence risk testing should be considered alongside shared decision-making, to determine the benefit of adjuvant chemotherapy. (Moderate Recommendation)	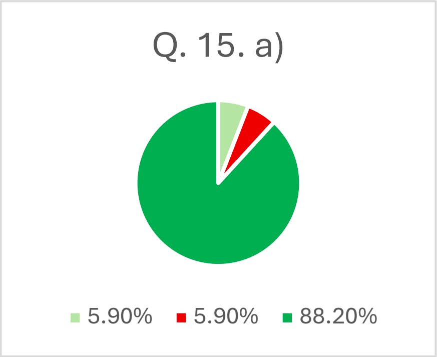<br>N=17	2nd round
15. b)	For patients with T1a, N0, HR+/HER2- EBC, recurrence risk testing should not be done. (Strong Recommendation)	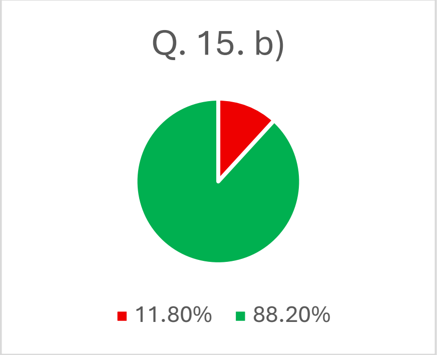<br>N=17	3rd round

16. a)	For patients with cN+, HR+/HER2– EBC where chemotherapy is indicated (neoadjuvant or adjuvant), a taxane-based regimen +/- anthracycline is recommended based on clinical risk, comorbidities, and shared decision-making. (Strong Recommendation)	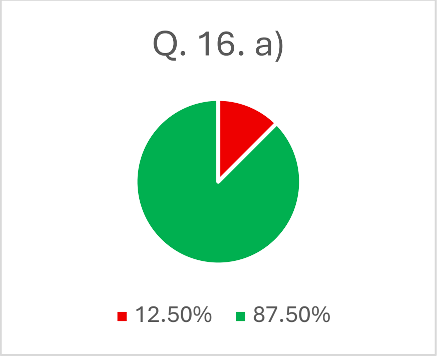 1 abstain<br>N=17	2nd round
16. b)	For patients with cN+, HR+/HER2– EBC with cardiac or other contraindications to anthracyclines, concerns about long-term toxicity, and/or who decline anthracycline use after shared decision-making, a non-anthracycline-based regimen (e.g., docetaxel + cyclophosphamide) is standard of care. (Strong Recommendation)	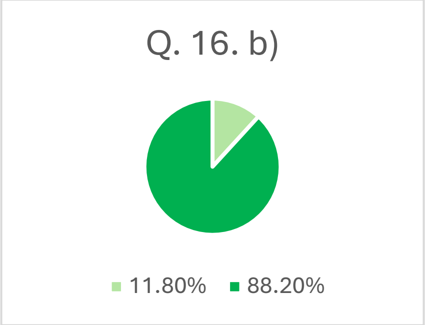	2nd round
17.	For premenopausal women with N0, HR+/HER- EBC at low risk of recurrence, standard of care treatment is ET. (Strong Recommendation)	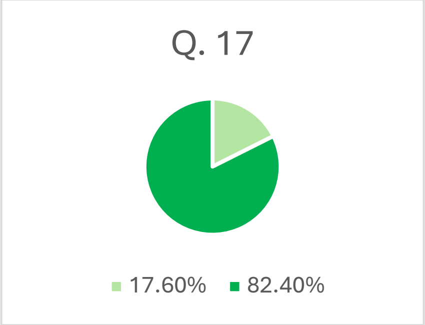	2nd round

18.	For premenopausal women with HR+/HER2– EBC at high risk of recurrence, standard of care treatment is adjuvant ET + CDK4/6i, as selected through shared decision-making. (Strong Recommendation)	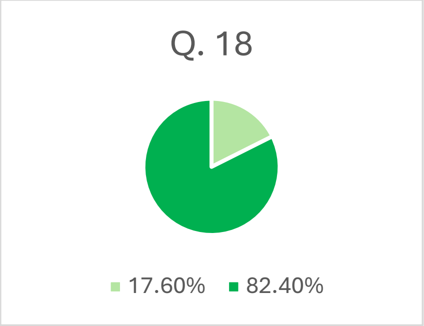<br>N=17	2nd round
19.	For postmenopausal women with HR+/HER2– EBC at low risk of recurrence, standard of care treatment is ET. (Strong Recommendation)	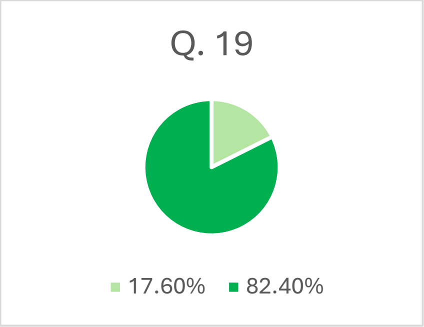<br>N=17	2nd round
20.	For postmenopausal women with HR+/HER2– EBC at high risk of recurrence, standard of care treatment is adjuvant ET + CDK4/6i as selected through shared decision-making. (Strong Recommendation)	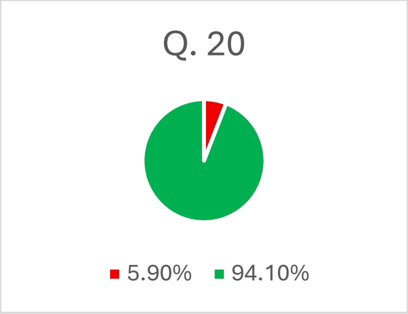<br>N=17	3rd round

21.	For patients with T2N0, HR+/HER2– EBC and high-risk features (i.e., Ki-67 ≥20%, Grade 3 histology, or high genomic risk score), standard of care treatment is adjuvant AI + 3 years of ribociclib through shared decision-making. (Strong Recommendation)	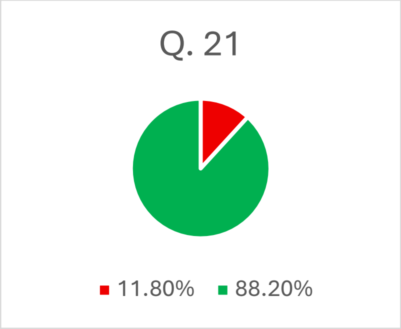<br><br>N=17	3rd round
22.	For patients with HR+/HER2– EBC who have been on ET + CDK4/6i for at least 6 months and are stable, clinical and laboratory monitoring can be done less frequently than monthly. (Strong Recommendation)	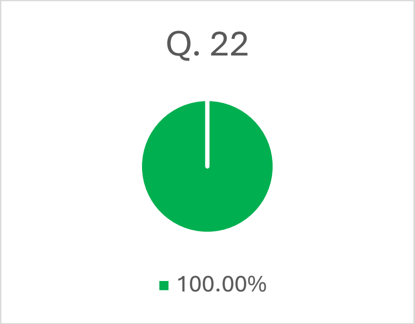<br><br>1 abstain<br>N=17	3rd round
23.	For patients with HR+/HER2– EBC receiving ET + CDK4/6i, management by an expert breast cancer healthcare professional practicing within an established monitoring pathway providing patient education, adherence support, drug/drug interaction assessment, side effect management, and blood work monitoring is standard of care. (Strong Recommendation)	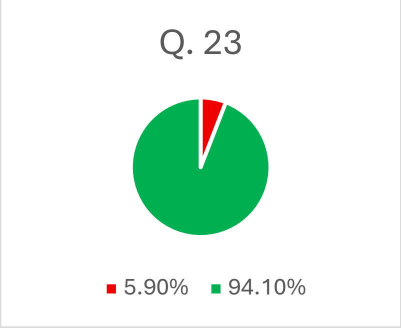<br><br>N=17	3rd round

24.	For patients with HR+/HER2– EBC and a germline BRCA1/2 pathogenic variant at high risk of recurrence (as per OlympiA trial criteria), standard of care treatment is olaparib for 1 year, followed by consideration for a CDK4/6i. (Strong Recommendation)	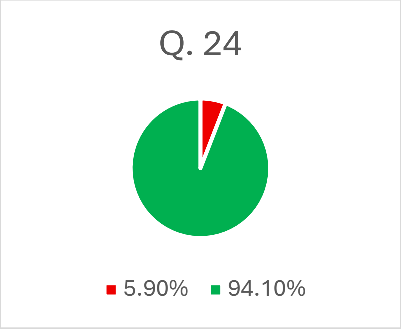 N=17	3rd round
25.	For patients with ER-low (1-10%) HR+/HER2– EBC, adjuvant ET +/- CDK4/6i could be discussed, though the absolute benefit is lower compared to more strongly ER+ tumours. (Moderate Recommendation)	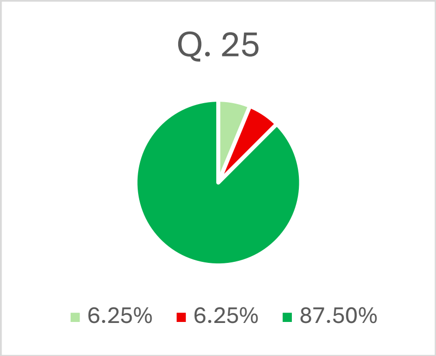 1 abstain<br>N=17	2nd round
26.	For postmenopausal women, or premenopausal women rendered postmenopausal, who are at higher risk of recurrence, bisphosphonates are standard of care to reduce the risk of bone metastases. (Strong Recommendation)	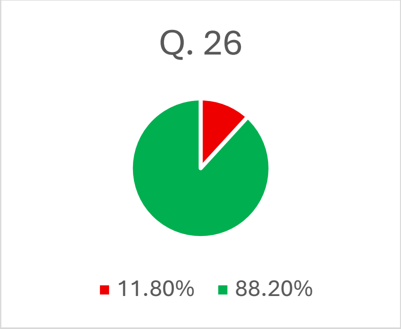 N=17	3rd round

27.	For any patient of child-bearing potential with HR+/HER2– EBC, the standard of care is to discuss and provide information on family planning and fertility preservation options before treatment. (Strong Recommendation)	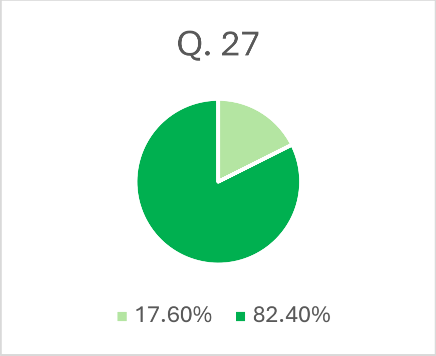 N=17	2nd round
28.	For patients with HR+/HER2– EBC who are pregnant, consultation with relevant multidisciplinary specialists is the standard of care. (Strong Recommendation)	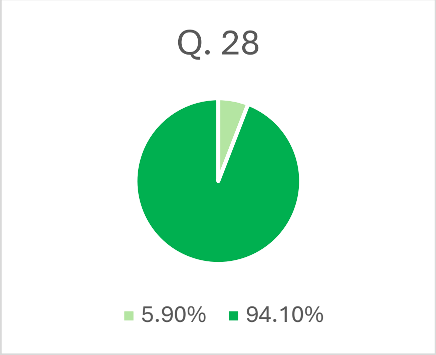 N=17	2nd round
